# *De novo* chromosome‐level genome of a semi‐dwarf cultivar of *Prunus persica* identifies the aquaporin *PpTIP2* as responsible for temperature‐sensitive semi‐dwarf trait and *PpB3‐1* for flower type and size

**DOI:** 10.1111/pbi.13767

**Published:** 2022-01-04

**Authors:** Xiaodong Lian, Haipeng Zhang, Chao Jiang, Fan Gao, Liu Yan, Xianbo Zheng, Jun Cheng, Wei Wang, Xiaobei Wang, Xia Ye, Jidong Li, Langlang Zhang, Zhiqian Li, Bin Tan, Jiancan Feng

**Affiliations:** ^1^ College of Horticulture Henan Agricultural University Zhengzhou China; ^2^ Henan Key Laboratory of Fruit and Cucurbit Biology Zhengzhou China

**Keywords:** Peach (*Prunus persica*), high‐quality genome assembly, genome‐wide association studies, temperature‐sensitive semi‐dwarf, flower type and size, fruit hairiness/hairless, *F‐M* locus

## Abstract

Peach (*Prunus persica*) is one of the most important fruit crops globally, but its cultivation can be hindered by large tree size. ‘Zhongyoutao 14’ (CN14) is a temperature‐sensitive semi‐dwarf (*TSSD*) cultivar which might be useful as breeding stock. The genome of CN14 was sequenced and assembled *de novo* using single‐molecule real‐time sequencing and chromosome conformation capture assembly. A high‐quality genome was assembled and annotated, with 228.82 Mb mapped to eight chromosomes. Eighty‐six re‐sequenced F_1_ individuals and 334 previously re‐sequenced accessions were used to identify candidate genes controlling *TSSD* and flower type and size. An aquaporin tonoplast intrinsic protein (*PpTIP2*) was a strong candidate gene for control of *TSSD*. Sequence variations in the upstream regulatory region of *PpTIP2* correlated with different transcriptional activity at different temperatures. *PpB3‐1*, a candidate gene for flower type (*SH*) and flower size, contributed to petal development and promoted petal enlargement. The locus of another 12 agronomic traits was identified through genome‐wide association study. Most of these loci exhibited consistent and precise association signals, except for flesh texture and flesh adhesion. A 6015‐bp insertion in exon 3 and a 26‐bp insertion upstream of *PpMYB25* were associated with fruit hairless. Along with a 70.5‐Kb gap at the *F‐M* locus in CN14, another two new alleles were identified in peach accessions. Our findings will not only promote genomic research and agronomic breeding in peach but also provide a foundation for the peach pan‐genome.

## Introduction

Peach (*Prunus persica* L.), a member of the Rosaceae family, is one of the most important deciduous fruit trees in the world. The harvested area was 1,527,052 ha globally in 2019 (www.fao.org). Peach originated about 2.47 million years ago in southwest China in glacial refugia generated by the uplift of the Tibetan plateau (Yu *et al*., 2018). Archaeological evidence shows that peach was planted at least 7500 years ago in China (Zheng *et al*., 2014). Peach trees were introduced from China to other parts of Asia, Europe and the Americas.

In 2013, the first version of the peach genome (Peach v1.0) was published (International Peach Genome, 2013), and a new version (Peach v2.0) was released in 2017 (Verde *et al*., 2017). Both versions were generated from the doubled haploid cultivar Lovell (PLOV2‐2N). The availability of these genomic datasets led peach research into the post‐genomic era (Cao *et al*., 2016; Dardick *et al*., 2013; Gu *et al*., 2016; Hollender *et al*., 2016; Pan *et al*., 2015; Pirona *et al*., 2013; Vendramin *et al*., 2014). The re‐sequencing of 10 wild and 74 cultivated peach varieties, followed by population analysis, revealed that a single domestication event occurred during peach evolution (Cao *et al*., 2014). During this domestication, selection for fruit size emerged prior to the selection for fruit skin colour (Yu *et al*., 2018). There were distinct phases of domestication and improvement, which led to increases in fruit size and changes in flavour (Li *et al*., 2019).

In peach, whole‐genome re‐sequencing has been an efficient approach for large‐scale marker development for mapping traits. A genome‐wide association study (GWAS) for 12 key agronomic traits was performed using whole‐genome re‐sequencing data of 129 peach accessions, in which nine qualitative traits showed consistent and more precise association signals than previously detected by linkage analysis (Cao *et al*., 2016). Flower type was mapped to scaffold 8 (Cao *et al*., 2016), which was consistent with a previous study (Fan *et al*., 2010). Flower type is controlled by a single gene with two alleles and is divided into two classes, showy (*sh*) and non‐showy (*SH*) (Bailey and French, 1942). Showy flowers are larger, while non‐showy flowers are medium or small in size (Bailey and French, 1942). However, flower size is controlled by multiple genes (Bailey and French, 1942) that remain to this day unmapped and unidentified.

In another study, several quantitative traits, such as fruit weight, sorbitol content and polyphenol levels were evaluated by GWAS (Cao *et al*., 2019). Re‐sequencing‐based Quantitative Trait Loci (QTL) or Mendelian Trait Loci (MTL) identified several candidate genes, such as the MTLs for dwarf (*dw*) (Hollender *et al*., 2016) and broom (*br*) (Dardick *et al*., 2013), and the QTL for maturity date (Pirona *et al*., 2013). These genome‐wide approaches improve the efficiency and accuracy of mapping through use of high‐density SNPs or markers and have provided valuable information for marker‐assisted selection (MAS) in peach breeding.

The re‐sequencing of hundreds of wild and cultivated peach genomes combined with various complexity reduction and genotype‐by‐sequencing strategies have enriched the pool of sequence information available for peach. However, all of the re‐sequenced genomes and subsequent processing strategies were done in comparison with the genomic sequences of the peach rootstock Lovell. Lovell is a double haploid that appears to be homozygous. Almost all cultivated peach varieties are heterozygous and are propagated clonally. This heterozygosity contributed to phenotypical impacts during domestication (Fournier‐Level *et al*., 2011). The use of Lovell as the base template may result in bias due to significant differences in genome structure between this rootstock and the different varieties of peach. Many genome‐specific loci were annotated to contain genes, suggesting that potential biological characteristics would be missed by standard reference‐mapping approaches. For example, a 36,320‐bp insertion in the tropical maize inbred line SK (*Zea mays*) contained three expressed genes that were not present in B73 (Yang *et al*., 2019). In rice (*Oryza sativa*), many genome‐specific loci were detected among three divergent varieties using whole‐genome alignment, with several of the loci associated with agriculturally important traits (Schatz *et al*., 2014). According to research in other crops, a single reference genome cannot account for all the genes of a species (Xie *et al*., 2019). A high‐quality *de novo* genome assembly is beneficial to the elucidation of the basis of phenotypic diversity. For example, a large inversion perfectly co‐segregates with flat‐fruit shape in peach (Guan *et al*., 2021; Guo *et al*., 2020; Zhou *et al*., 2019).

A complete, accurate and high‐quality genome provides a powerful foundation for identifying candidate genes, such as seen in apple (*Malus* × *domestica*) (Zhang *et al*., 2019), maize (*Zea mays*) (Yang *et al*., 2019) and strawberry (*Fragaria nilgerrensis*) (Zhang *et al*., 2020). Using high‐quality genome assembly, a specific LTR retrotransposon, associated with red skin in apple, was identified in the genome of the homozygous line HFTH1 but not in the GDDH13 genome (Zhang *et al*., 2019). At this point, more than one *de novo* genome has been released for humans (Zhang *et al*., 2021) and many plants like *Arabidopsis thaliana* (Michael *et al*., 2018; The Arabidopsis Genome, 2000), rice (Schatz *et al*., 2014) and maize (Schnable *et al*., 2009; Springer *et al*., 2018; Sun *et al*., 2018). The sequencing of multiple genomes provides a huge amount of information that is crucial to further studies on countless agronomically important traits.

One critical area of trait discovery is how plants respond to environmental issues such as global warming. Global warming now commands worldwide attention, especially as 2015–2020 were the six warmest years on record. If climate change is not addressed, by 2050 crop yields are predicted to decrease by 25% (http://www.fao.org). This creates an urgent need to elucidate the molecular mechanisms by which plants adapt to elevated temperatures. Many studies on temperature perception and signalling have been performed, mainly by exploiting the model plant Arabidopsis (Quint *et al*., 2016). The phytochrome B‐phytochrome interacting factor 4‐auxin (phyB‐PIF4‐auxin) module is one component of temperature sensing (Casal and Balasubramanian, 2019). However, little is known about other participants in temperature sensing. Temperature affects overall growth and productivity of many crops, but can also specifically influence certain traits when these traits are regulated by specific genes.

Most cultivated peach varieties are standard sized, with high branch architecture and a large amount of annual branch growth. This creates a great deal of manual labour for pruning or application of phytohormones during cultivation and production in commercial orchards. How to alter plant’s architecture easily and efficiently is a great challenge to scientists. The peach cultivar ‘Zhongyoutao 14’ (CN14) was derived from SD9238 and has a temperature‐sensitive semi‐dwarf (*TSSD*) phenotype. The internode length is temperature dependent, with extremely shortened internodes at temperatures below 30 °C and normal internode length above 30 °C (Lu *et al*., 2016). The mechanism regulating temperature‐sensitive shoot elongation in CN14 might be different than that of the phyB‐PIF4‐auxin module (Lian *et al*., 2020). In the only published study on this trait, the *TSSD* locus was mapped to a region spanning approximately 750 kb between 2.35 and 3.10 Mb on scaffold 3 based on the Lovell genome v1.0 (Lu *et al*., 2016). Precise identification of the gene(s) controlling the temperature‐sensitive semi‐dwarf trait in peach will provide not only a valuable resource for revealing the mechanisms by which plants sense temperature but also their potential use in the genetic improvement of peach for labour‐saving cultivation.

Here, we present a high‐quality genome of CN14 using high‐depth PacBio long‐read data complemented with Illumina short‐read data and Hi‐C sequencing. The locus or candidate genes of *TSSD* and flower type (non‐showy, *SH*) were identified using three strategies, while another 13 agronomic traits were identified based on the new high‐quality genome using GWAS. The CN14 genomic sequences will provide a valuable resource for further molecular functional analyses and comparative genomics research across the Rosaceae family.

## Results

### Genome sequencing, assembly and assessment of CN14

**Figure 1:**
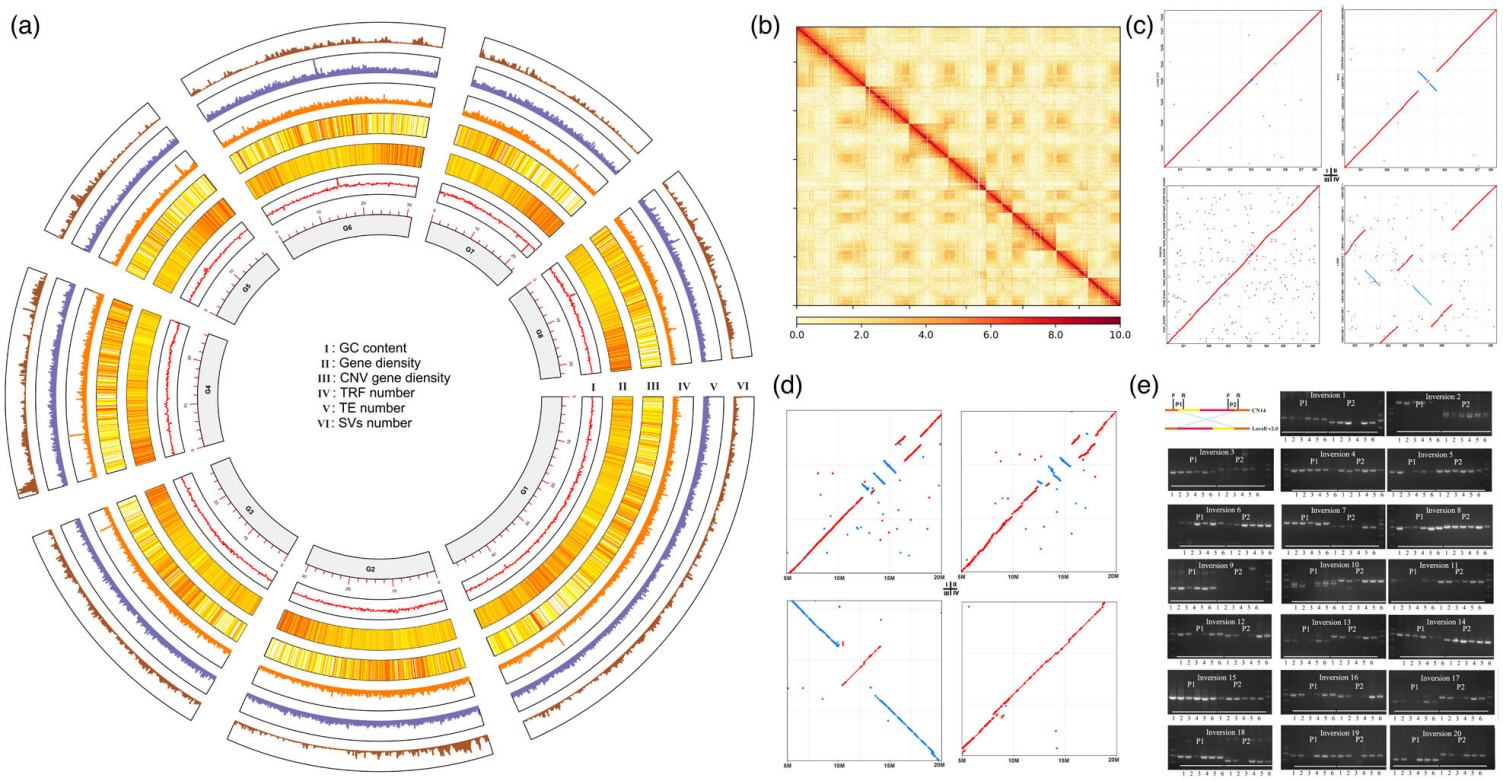
Characterization of the CN14 genome. (a), Circos plot shows GC content, gene density, CNV gene density, TRF number, TE number and SVs number. (b), Hi‐C interactions among eight chromosomes. Strong interactions are indicated in dark red and weak interactions are indicated in yellow. (c), Collinearity of genome of CN14 versus Lovell v2.0 (I), CN14 versus RYP1 (II), CN14 versus 124Pan (III) and CN14 versus LHSM (IV). (d), Collinearity block of Chr3: 5–20 Mb. (e), Identification of 20 large inversions on Chr3 in six peach accessions. P1: Primer pair at left end of inversion. P2: Primer pair at right end of inversion. 1: Okubo; 2: Matsumori; 3: Fenshouxing; 4: Phillips; 5: CN14; and 6, HSM. The positions of inversions were listed in Table S14.

Together, the three sequencing strategies generated ~586‐fold coverage of the peach genome using data from the PacBio Sequel technology (161.30 Gb with an average length of 21.7 Kb), 165‐fold from DNBseq short reads (39.68 Gb) and 550‐fold from the Hi‐C data (132.24 Gb). PacBio long reads were used to assembly the genome using CANU (v1.9), creating a contig‐level genome containing 236.58 Mb (186 contigs; Table 1). The contig N50 was 6.86 Mb, and the longest contig was 14.05 Mb. The Hi‐C data, yielding 881.63 million paired‐end reads, were used to anchor the contigs to chromosomes using Hic‐Pro software, to generate a chromosome‐level genome containing 228.82 Mb (96.74%) mapped on eight chromosomes with a scaffold N50 of 27.86 Mb. The longest scaffold was 48.77 Mb (Table 1 and Figure 1a,b). Based on a k‐mer analysis (k = 23), the CN14 peach genome had a heterozygosity rate of 0.31% and GC content was 37.9% (Figure 1a and Figure S1).

**Table 1:** Summary of features of the assembled CN14 peach genome

Assembly feature	CN14*	Lovell v2.0^†^
Total assembly size (Mb)	236.53	227.4
Largest scaffolds (Mb)	48.77	47.85
Scaffold N50 (Mb)	27.89	27.37
Number of contigs	186	2525
Largest contig (Mb)	14.1	1.5
Contig N50 (K bp)	6866.8	255.4
Gaps (%)	0.015	1.22
Sequence anchored to chromosomes (Mb)	228.82	225.7
Repeat regions of assembly (%)	46.45	44.85
Predicated gene models	30181	31972
Complete BUSCO (%)	98.0	97.6
LTR assembly index (LAI)	24.51	21.29

* Summary of features of CN14 genome in this study.

^†^ Lovell v2.0 genome was referenced from previous studies (Verde *et al*., 2017).

The quality of the assembled CN14 genome was assessed using the LTR Assembly Index (LAI) (Ou *et al*., 2018) and Benchmarking Universal Single‐Copy Orthologs (BUSCO, v3) (Manni *et al*., 2021). The LAI score was 24.51, a ‘gold standard level’ that exceeded that of the Lovell v2.0 genome (LAI of 21.29) (Table 1). The BUSCO method was used to assess the completeness of the genome (98%) and protein sequence (99%), with both scores representing complete BUSCO annotations (Table S1). LAI and BUSCO results indicated that the CN14 genome was a high‐quality genome.

### Genome annotation of CN14

Approximately 46.45% of the CN14 genome was annotated as repeat sequences, including 38% listed as transposable elements (TEs) (Figure 1a and Table S2). Based on the RNA‐seq data, *de novo* predication and homology‐based search, 30181 protein‐coding genes were identified in the CN14 genome. The predicted protein‐coding genes were then annotated by comparing to the COG, GO, eggNOG, KEGG, NR, SwissPort, Rice and Tair databases. In total, 98.11% of the coding genes were functionally annotated (Figure 1a and Table S3).

### Genomic variation between the CN14 and four other peach genomes

The CN14, Lovell v2.0, 124Pan, RYP1 and Longhuashuimi (LHSM) peach genomes were compared using the MUMmer program. The genomes showed excellent collinearity, except for chromosome 3 (Chr3) (Figure 1c). Approximately 98.67% (211.97 Mb) of the CN14 genomic sequences matched one‐to‐one syntenic blocks with 98.98% (210.90 Mb) of the Lovell v2.0 genome. Assemblytics analysis revealed 75,210 variants between the CN14 and Lovell v2.0 genomes, with a total length of 5,697,068 bp including 3530 structural variations (SVs, >50 bp). Among the SVs, there were 805 insertions, 646 deletions, 844 repeat contractions, 909 repeat expansions, 57 tandem contractions and 269 tandem expansions (Figure 1a, Table S4 and Table S5).

There were 97 large SVs (>10 Kb) in the 12–17 Mb region of Chr3, including five large inversions (Figure 1d‐I and Table S6). Other large inversions were observed between CN14 and RYP1 (Figure 1d‐II) and between CN14 and 124Pan (Figure 1d‐III), but not between CN14 and LHSM (Figure 1d‐IV). To further identify the SVs in this regions, 20 large SVs were confirmed in CN14 and other five cultivars using PCR. The results showed that most of these SVs were present in six peach germplasms (Figure 1e).

### Evolution and gene family expansion/contraction

**Figure 2:**
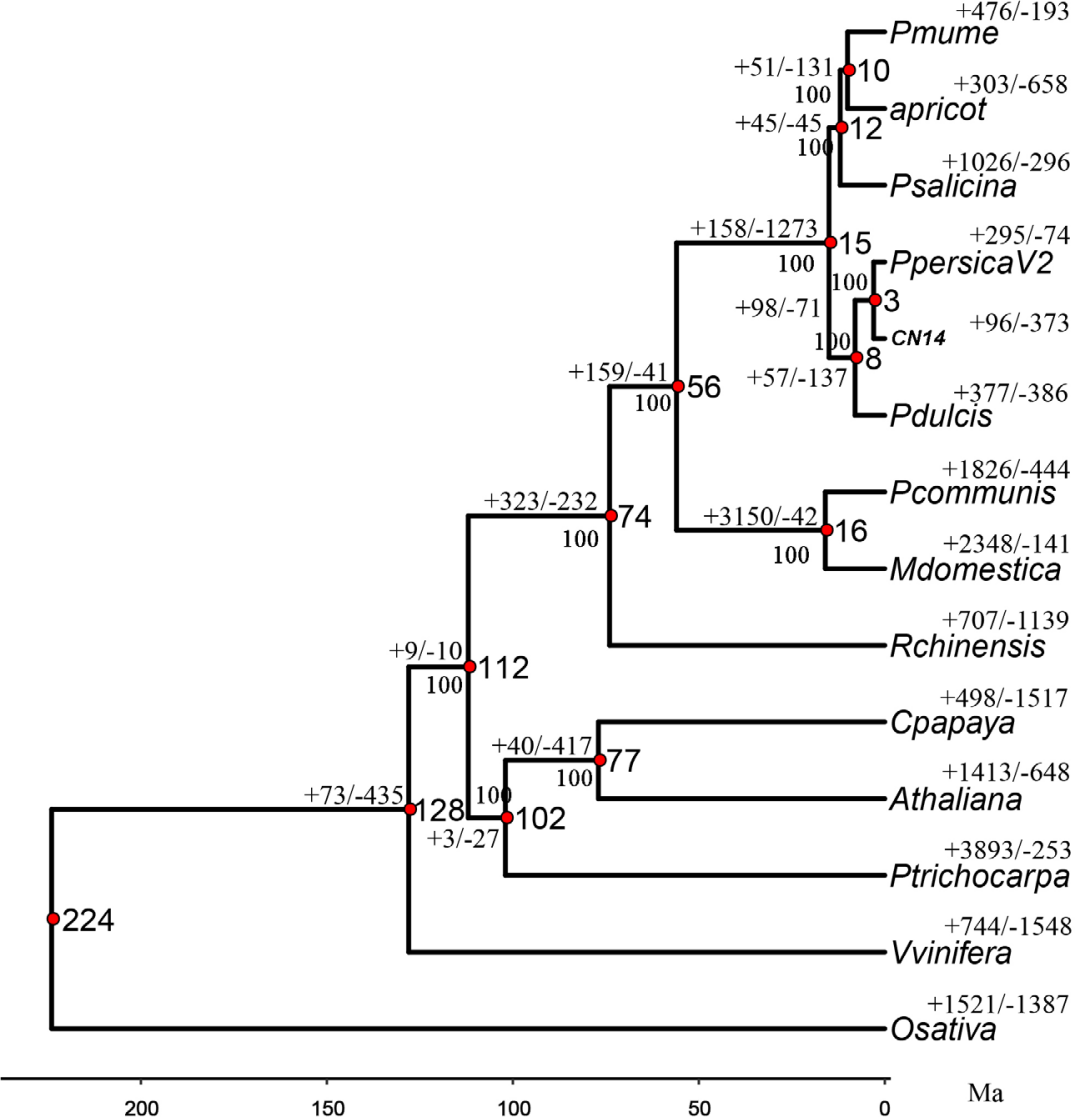
The estimation of divergence time and expansion or contraction of gene families in *Prunus mume*, *Prunus apricot*, *Prunus salicina*, *Prunus persica* v2.0, CN14 (*Prunus persica* L.), *Prunus dulcis*, *Prunus communis*, *Malus* × *domestica*, *Rose chinensis*, *Carica papaya*, *Arabidopsis thaliana*, *Populus trichocarpa*, *Vitis vinifera* and *Oryza sativa*.

The genomes of CN14 and Lovell peach (v2.0) and 12 other plant species, including seven species in Rosaceae (*P. mume*, *P. apricot*, *P. salicina*, *P. dulcis*, *Pyrus communis*, *Malus* × *domestica* and *Rosa chinensis*), four eudicot species (*Arabidopsis thaliana*, *Populus trichocarpa*, *Carica papaya* and *Vitis vinifera*) and the monocot *Oryza sativa* as the outgroup, were compared (Figure 2). A high confidence phylogenetic tree of the 14 species was generated using genes extracted from 256 orthogroups from single‐copy gene families. As expected, CN14 and its close relatives in *Prunus* (*P. mume*, *P. apricot*, *P. salicina* and *P. dulcis*) were clustered into one monophyletic group, with the *P. persica* cultivars showing the closest relationships with *P. dulcis*. Compared with the other genomes, there were 96 expanded and 373 contracted orthogroups in the CN14 genome. The expanded orthogroups, including 1559 genes, were predicted to function mainly in protein activation, RNA processing and miscellaneous and secondary metabolism (Table S7 and Figure S2a). There were 3021 genes in the contracted orthogroups, mainly predicted to function in protein activation, RNA processing, miscellaneous, transport and secondary metabolism (Table S7 and Figure S2b). The specific flavour of peach fruit is mainly affected by primary and secondary metabolism. Interestingly, 21 sugar and 18 acid transport‐related genes were in the expanded or contracted orthogroups respectively. These orthogroups might contribute to peach flavour.

### An aquaporin tonoplast intrinsic protein 2 (*TIP2*) gene is responsible for temperature‐sensitive semi‐dwarf (*TSSD*) in peach

**Figure 3:**
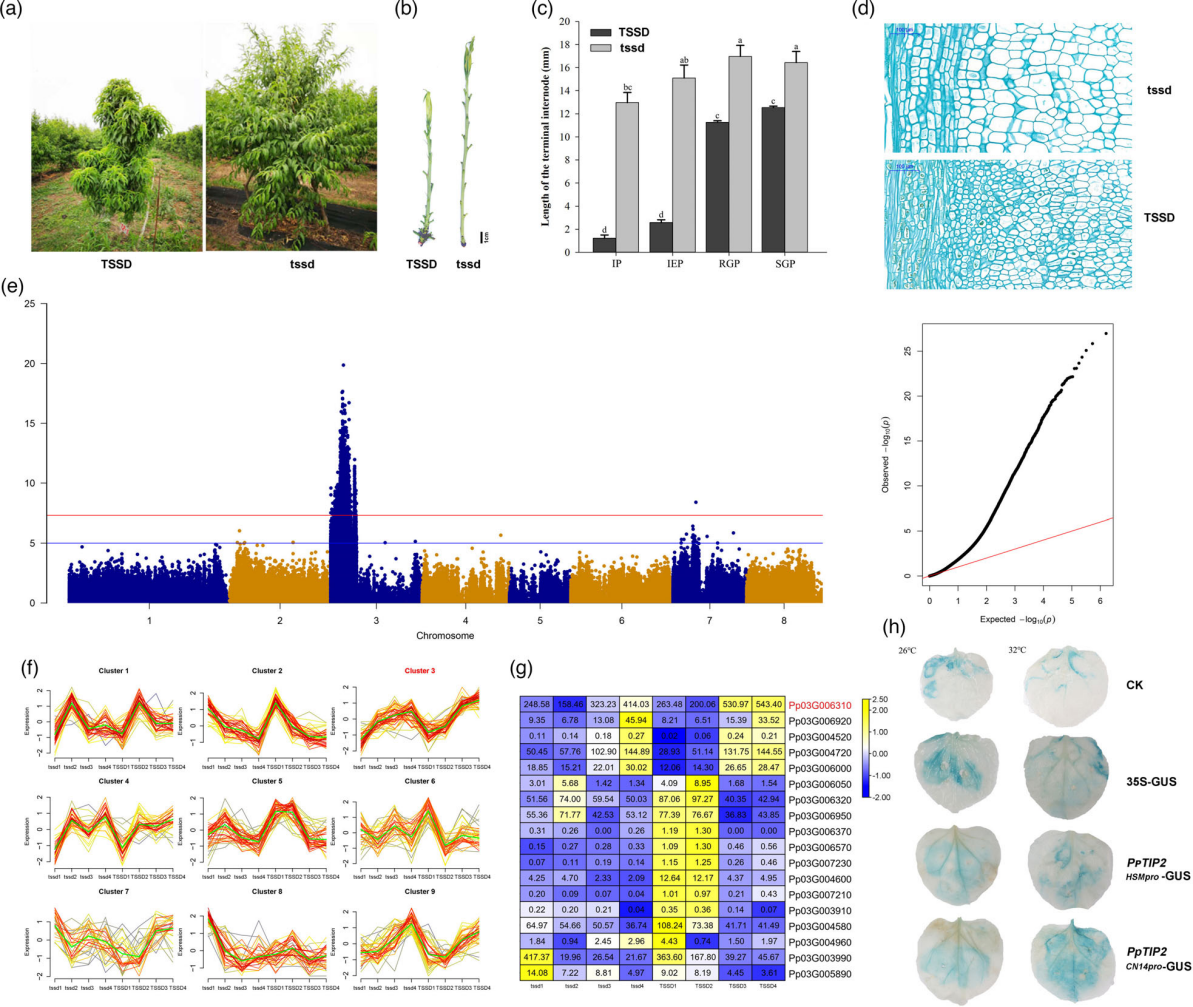
Identification of the gene underlying the temperature‐sensitive semi‐dwarf trait (*TSSD*). (a), Tree habit phenotype. (b), Shoot phenotype of *TSSD* and *tssd*. Bar = 1 cm. (c), Terminal internode length of CN14 (*TSSD*) and HSM (*tssd*) at four different stages: IP, initial period; IEP, initial elongation period; RGP, rapid growth period; and SGP, stable growth period. Different letters indicate significant divergence of the length using Duncan’s multiple range test (*P* < 0.05). (d), Cell length in the annual stem base from CN14 and HSM. (e), the *TSSD* locus was located on Chr3 using GWAS. (f), Expression pattern of genes in candidate region. TSSD1‐TSSD4 and tssd1‐tssd4 were CN14 and HSM sampled at IP, IEP, RGP and SGP stages respectively. (g), Expression level of DEGs in *TSSD* locus. (h), Activity of the *PpTIP2* promoters from CN14 and HSM at 26°C and 32°C.

The internode length of CN14 is extremely shortened at temperatures below 30 °C. To reveal the genetic basis of this temperature‐sensitive semi‐dwarf (*TSSD*) phenotype, a hybrid population from CN14 (*TSSD*) and HSM (*tssd*) was constructed, and 86 F_1_ individuals were obtained. Among them, 34 individuals were *TSSD*, while 52 were *tssd*, resulting in an observed 1:1 Mendelian ratio and fitting with the expected ratio for a monogenic dominant genetic control (χ^2^ = 3.76; *P* > 0.05) (Table S8). The tree height of *TSSD* was semi‐dwarf (Figure 3a) because of short basal internode length (Figure 3b). The lengths of the terminal internode of the two parents were measured at four critical growth stages (Figure 3c). The terminal internode of *TSSD* was shorter at both the initial period (IP) and the initial elongation period (IEP) (<3 mm in *TSSD* compared to >13 mm in *tssd*). The terminal internode was significantly longer at the RGP and SGP stages (11.27 mm and 12.54 mm, respectively), but still shorter than in *tssd*. In contrast, the length of the internode of *tssd* displayed little change among the four stages (12.97 mm, 15.10 mm, 16.97 mm and 16.44 mm at IP, IEP, RGP and SGP, respectively). The cell length of the annual stem base in *TSSD* was shorter than that of *tssd* (Figure 3d).

Three analyses were performed on the genome re‐sequencing data from 86 offspring of the CN14 × HSM cross to identify major loci contributing to the *TSSD* phenotype. First, allele frequency directional difference and density (AFDDD) mapping was performed according to Dougherty *et al*. (Dougherty *et al*., 2018). A 2‐Mb genomic region on Chr3 (3–5 Mb with peaks) showing a significant association with *TSSD* was revealed by conducting variant allele frequency and density‐based mappings (Figure S3). Second, the *TSSD* loci were mapped within a 0.24‐Mb (4.74–4.98 Mb) interval on Chr3 based on the genetic map and phenotypic data, with an LOD score of 9.02 that explained 30.31% of the variance (Figure S4). A total of 32 genes were predicted within this region. Thirdly, GWAS was carried out using the SNPs of 86 progeny and the genotypic data. The leading SNP candidate was Chr3: 4,376,637 (Figure 3e and Table 2).

**Table 2:** Significant association between agronomic traits and candidate genes

Trait	Chromosome	Position	*P*‐value	Candidate genes and functional annotation
Growth habit (*TSSD*/*tssd*)	G3	4,376,637	1.07E‐27	Pp03G006310 (TIP2)
Flower type (*SH*/*sh*)	G8	14,438,628	4.93E‐109	Pp08G015510 (B3‐1)
Flower size	G8	14,983,191	8.41E‐10	Pp08G015510 (B3‐1)
Flesh colour around stone	G4	11,063,696	4.81E‐14	Pp04G016610 (UDP‐Glycosyltransferase superfamily protein), Pp04G016630 (UDP‐Glycosyltransferase superfamily protein), Pp04G017800 (NAC6), Pp04G017810 (NAC19)
	G3	18,905,188	1.67E‐08	Pp03G021030 (MYB32), Pp03G021610 (MYB114), Pp03G021660 (MYB10)
	G8	19,929,060	1.65E‐08	Pp08G024560 (MYB‐like)
	G6	13,088,613	3.72E‐08	Pp06G024190 (WRKY)
Fruit maturity date	G4	11,019,990	1.18E‐13	Pp04G017800 (NAC6), Pp04G017810 (NAC19), Pp04G016770 (NAC77)
Fruit hairiness	G5	16,757,982	2.91E‐84	Pp05G004700 (MYB25)
Double flowers	G2	24,849,526	1.23E‐41	Pp02G0287000 (AP2)
	G6	19,298,780	3.60E‐41	Pp06G011800 (TOE type transcription factor)
Flesh colour (While/yellow)	G1	27,076,667	1.75E‐44	Pp01G032380 (CCD4)
Flower opening time	G1	27,195,361	8.51E‐11	Pp01G032380 (CCD4)
Pollen fertility	G6	1,944,951	4.89E‐21	Pp06G039250 (ATP synthesis‐related gene), Pp06G038900 (ATP‐binding cassette transporter G26)
Kernel taste	G2	27,148,186	1.10E‐39	Pp02G031770 (Anthranilate N‐benzoyltransferase protein), Pp02G031580 (Flavonol synthase/flavanone 3‐hydroxylase) and Pp02G032190 (L‐ascorbate oxidase precursor)
	G8	3,275,852	3.62E‐42	Pp08G004140 (NAC67), Pp08G004150 (NAC74)

To further identify which candidate gene is responsible for the *TSSD* trait, transcriptome analyses of four different growth periods (IP, IEP, RGP and SGP) of CN14 and HSM were performed. All expressed genes were grouped into nine clusters (Figure 3f), according to the phenotype. Cluster 3 showed a strong correlation with terminal internode length. For the *TSSD* phenotype at different development stages, IP and IEP were the slow elongation period, while RGP and SGP were the rapid elongation period. Comparison of the genes expressed in the later stages (RGP and SGP) and the primary stages (IP and IEP) revealed a total of 1044 differentially expressed genes (IP vs. RGP, IP vs. SGP, IEP vs. RGP and IEP vs. SGP; DEGs significance at *P* < 0.05). Among these DEGs, only 18 genes were located on Chr3: 3–5 Mb region (Figure 3g). The genes Pp03G006310, Pp03G006920, Pp03G004520, Pp03G004720 and Pp03G006000 showed lower transcript levels at the primary stages (IP and IEP) than at the later stages (RGP and SGP), an expression profile that correlates with shoot internode length. The transcript level of Pp03G004520 showed very low abundance, so was not considered. The gene Pp03G006310 showed the highest expression level and was annotated as an aquaporin, tonoplast intrinsic protein (TIP2) related to cell expansion. The qRT‐PCR results indicated that Pp03G006310 was highly expressed at the RGP and SGP stages (Figure S5). *PpTIP2* was considered a candidate gene that could contribute to internode development in CN14.

Within the *PpTIP2‐*coding region, there were no predicted amino acid polymorphisms for the PpTIP2 protein between the HSM and CN14. Interestingly, the promoter sequence of *PpTIP2* had two genotypes, which contains 33 variations (Table S9), with a heterozygous genotype (0/1) in CN14, while homozygous in HSM (1/1). Furthermore, 20 individuals showing *TSSD* and *tssd* were randomly selected to verify the genotype of *PpTIP2* promoter using Sanger sequencing respectively. The variations were co‐separated with the *TSSD* phenotype (Table S10). Some of the variations in the promoter were predicted to change five TF‐binding sites, including Dof, MADS, NAC and two C2H2 transcription factors (Figure S6). To identify the two prompter genotypes of *PpTIP2* response to different temperature, two promoters were used to construct *PpTIP2‐HSM_pro_‐GUS* and *PpTIP2‐CN14_pro_‐GUS* vector, then were expressed in *N. benthamiana* at 26 °C and 32 °C respectively. The GUS activity was higher at 32 °C than that at 26 °C in *PpTIP2‐CN14_pro_‐GUS*, while there was no difference between 32 °C and 26 °C in *PpTIP2‐HSM_pro_‐GUS* (Figure 3h). This suggested that the sequence variations in the upstream regulatory region of *PpTIP2* resulted in the low expression level of *PpTIP2* during the slow elongation period (IP and IEP), which may be responsible for the temperature‐sensitive semi‐dwarf phenotype.

### A SNP in *PpB3‐1* was highly associated with flower type and size

**Figure 4:**
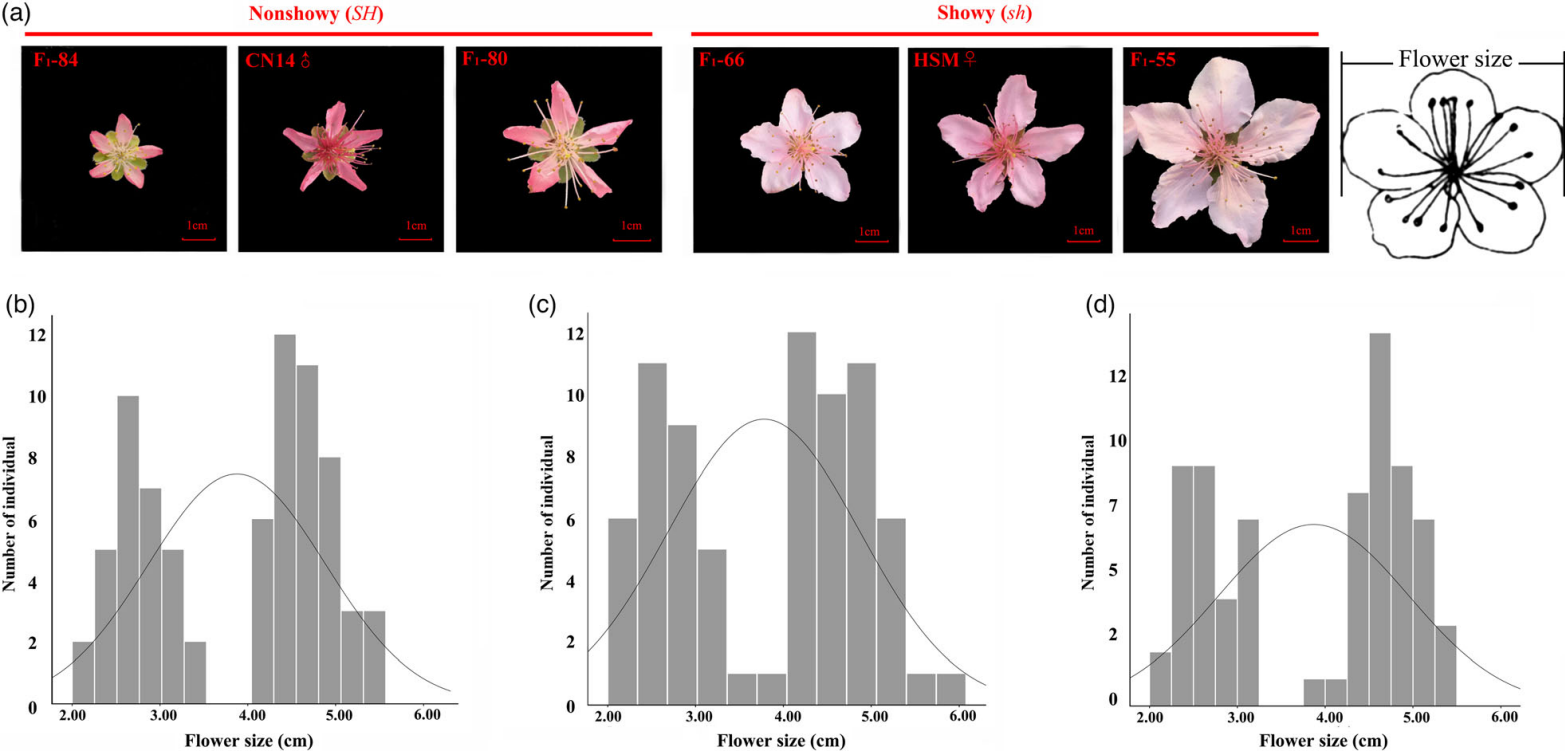
Phenotype and frequency distribution of flower type and size. (a) Phenotype of non‐showy and showy flowers of the two parents and four F_1_ progeny. (b, c and d) The frequency of flower size in the F_1_ progeny derived from HSM (*sh*) and CN14 (*SH*) in the years 2018, 2019 and 2020 respectively.

Of the 86 F_1_ individuals, 41 had the non‐showy (*SH*) flower phenotype and 45 were showy (*sh*) (Figure 4a and Table S8). The *Chi*‐squared test showed that flower type segregated at a ratio of 1:1 (χ^2^ = 0.017; *P* > 0.05), suggesting a monogenic inheritance of the trait (Table S8). The frequency of flower size was counted in 2018 (Figure 4b), 2019 (Figure 4c) and 2020 (Figure 4d). The flower size in CN14 (*SH*) was smaller, with annual averages of 2.84 cm, 2.76 cm and 2.88 cm (small), while HSM (*sh*) was larger, with annual averages of 4.93 cm, 5.02 cm and 5.10 cm (large) in 2018, 2019 and 2020 respectively. Flower size in the F_1_ segregating population showed continuous variation (ranging from 2.00 cm to 5.70 cm), indicating that the flower size is quantitatively inherited. The diameters of the showy flowers were always longer than 3.7 cm, while the non‐showy flowers were shorter than 3.7 cm.

**Figure 5:**
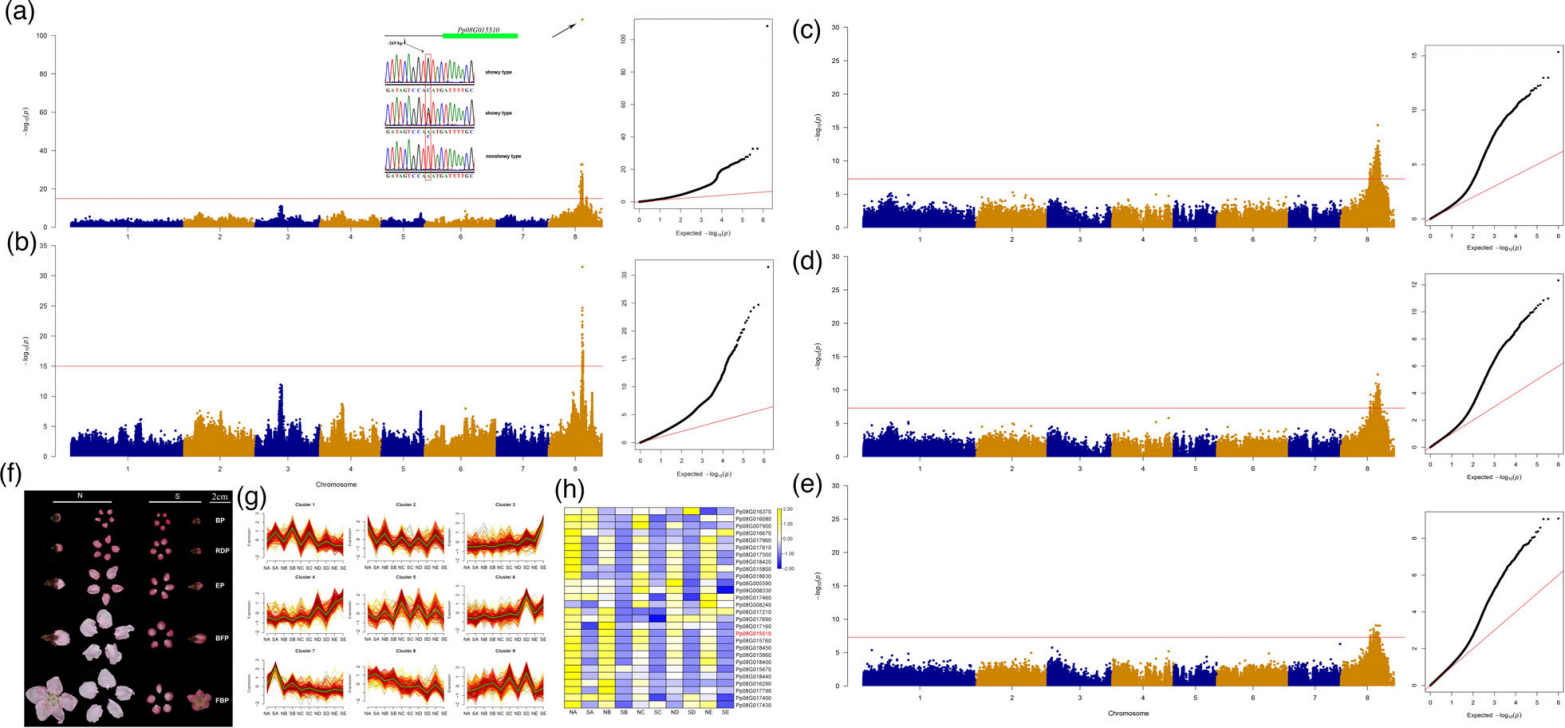
A candidate gene for flower type and size was located on Chr 8. (a), GWAS for flower type, a SNP with A/C mutation in the promoter of Pp08G015510 (*PpB3‐1*) was associated with showy (*sh*) and non‐showy (*SH*). (b), GWAS for flower type using 86 individuals in the F_1_ population (HSM × CN14). (c, d and e) GWAS for flower size in 2018, 2019 and 2020 respectively. (f), *SH* and *sh* flower phenotypes at five developmental stages: BP (buds period), RDP (red dot period), EP (equivalent in size between petal and sepal period), BFP (budding flower period) and FBP (full bloom period). (g), Expression pattern of candidate genes at the five stages. SA‐E and NA‐E were *sh* and *SH* sample at BP, RDP, EP, BFP and FBP of *SH* respectively. (h) DEGs within candidate region.

The *SH/sh* loci were located on Chr8 between 14 and 16 Mb based on the method of Dougherty *et al*. (Dougherty *et al*., 2018) (Figure S7). Here, the *SH/sh* gene was located in the region 15.66–16.36 Mb on Chr8, with an LOD score of 13.57 that explained 27.32% of the variance (Figure S8). Based on GWAS, a SNP (A‐C) located at 14,438,628 bp of Chr8 was highly associated with flower type (Figure 5a,b). This SNP locus was located in the promoter region of Pp08G015510 (*PpB3‐1*), which was predicted to encode a protein in the B3‐type transcription factor family (Figure 5a and Table 2). In addition, this locus was located at chr8: 14.7–16.4 Mb using GWAS based on flower size trait using 3‐year data (Figure 5c,d and e). Interestingly, the locus for flower size was the same as *SH/sh* (Table 2). These results suggested that *PpB3‐1* was a candidate gene for both flower type and flower size.

To further identify candidate genes for flower type and size, the petals of two flower types (*SH* and *sh*) at five developmental periods, namely BP (buds period), RDP (red dot period), EP (equivalent in size between petal and sepal period), BFP (budding flower period) and FBP (full bloom period) (Figure 5f), were analysed using RNA‐seq. The expression patterns of all genes could be classified into nine clusters (Figure 5g). Among the differentially expressed genes (DEGs) in the 200‐Kb candidate region, *PpB3‐1* showed higher transcript levels in the buds period (BP) in *sh* (Figure 5h). The qRT‐PCR results showed that *PpB3‐1* had a higher transcript level in the flower of *sh* than in *SH* (Figure S9). The result further indicated that *PpB3‐1* was the most likely candidate gene controlling flower type and flower size in this peach population.

For confirmation of the associated A/C SNP, 161 peach accessions (88 natural accessions and 73 hybrids from HSM and CN14) were further evaluated by PCR. In the natural population, all 55 showy accessions contained a homozygous A/A genotype, while the 33 non‐showy germplasms were heterozygous A/C (29) or homozygous C/C (4) (Table S11). In the hybrid population, 43 showy accessions had a homozygous A/A genotype, while 30 non‐showy germplasms were heterozygous A/C genotypes. All results indicated that the SNP variation in the promoter of *PpB3‐1* might contribute to flower type.

### GWAS‐based high‐quality genome to identify candidate genes for other agronomic traits

The high‐quality genomic sequence of CN14 was subjected to a GWAS with 334 other sequenced peach accessions for 12 agronomic traits (Table 2 and Table S12).

**Figure 6:**
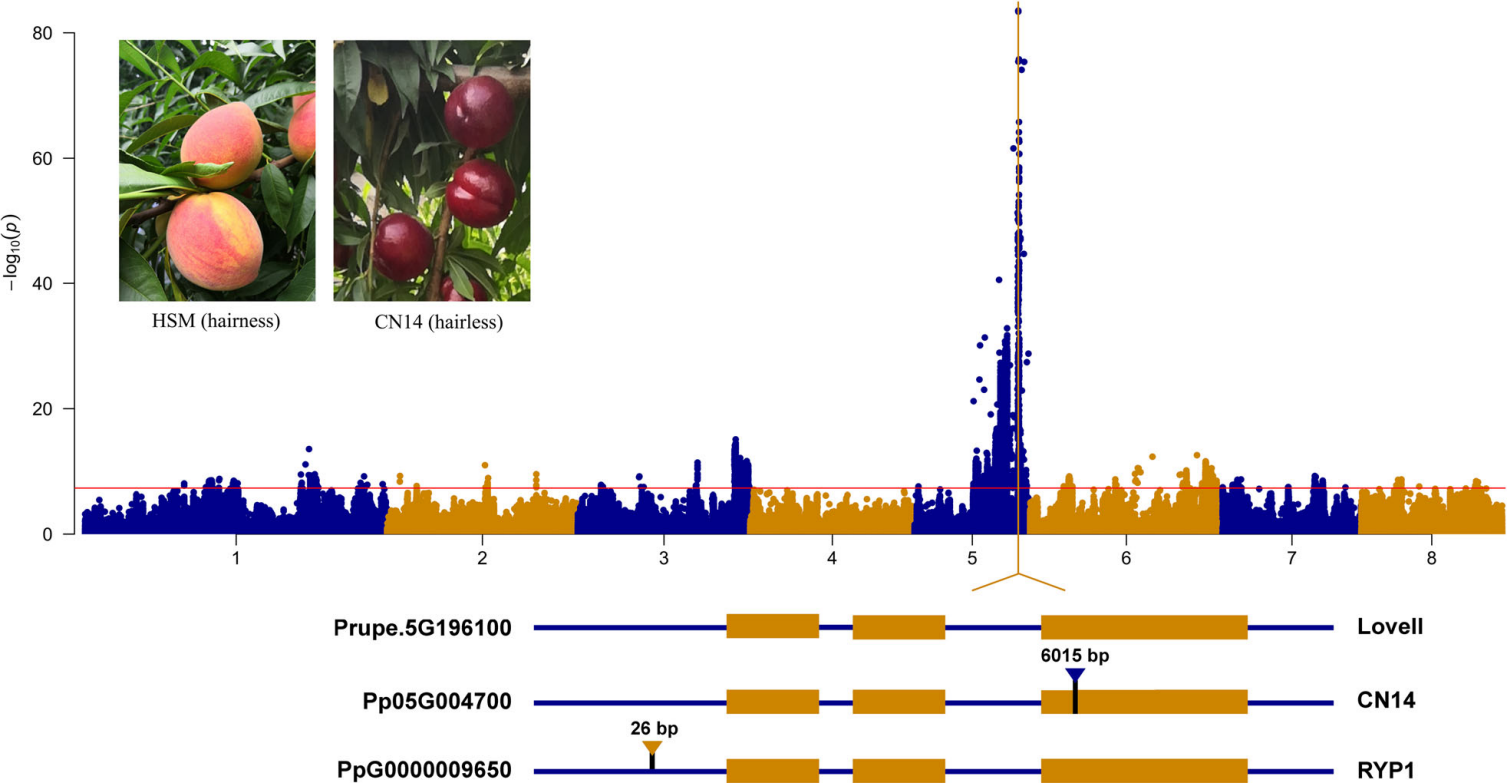
Genome‐wide association for fruit hairiness/hairless and different variants of *PpMYB25* in three genomes (Lovell, CN14 and RYP1). Compared with the sequence of *PpMYB25* in Lovell v2.0 genome, CN14 carried a 6015‐bp LTR retroelement insertion in exon 3 of the *PpMYB25* gene, and RYP1 carried a 26‐bp insertion upstream of *PpMYB25*.

The fruit hairiness/hairless trait is an important trait for peach peel, and hairless skin is one of the most remarkable and distinguishable traits. In a previous study using the Lovell v2.0 genome, the hairiness/hairless trait was mapped to Chr5:15,960,687, and the candidate gene was *PpMYB25* (Vendramin *et al*., 2014). However, the variation at this locus could not distinguish all hairiness or hairless cultivars in peach. In this study, a significant association peak was located at Chr5:16,757,982 of CN14 genome (Table 2 and Figure 6). Compared with the Lovell v2.0 genome, a 6015‐bp insertion in the third exon of Pp05G004700 (*PpMYB25*) was found in the CN14 (hairless) genome and in 60 other hairless accessions based on re‐sequencing data. To confirm that the variation at this locus is associated with the hairiness/hairless trait, 60 peach accessions (21 hairiness and 39 hairless) were subjected to targeted PCR. The results showed that 39 hairless peach accessions contained a homozygous insertion (*g*/*g*), while hairiness peach accessions were heterozygous (*G*/*g*) or had no insertion (*G*/*G*) (Figure S10 and Table S13). The RYP1 nectarine (hairless) genome did not carry this insertion in the *PpMYB25* gene, but it did contain a 26‐bp insertion upstream of *PpMYB25* (Figure 6).

**Figure 7:**
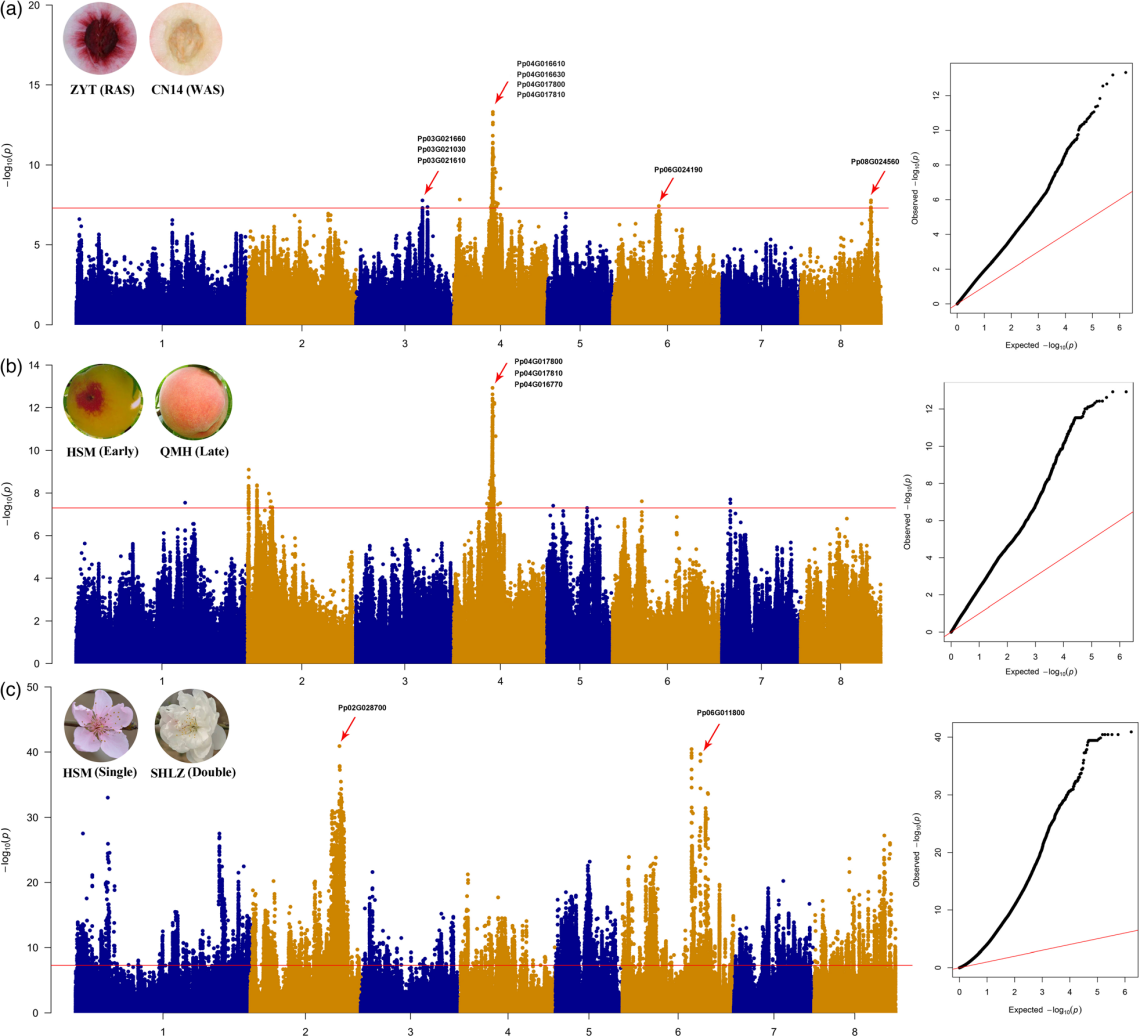
Identification of candidate genes for flesh colour around stone, fruit maturity date and double flower. (a), Candidate locus for flesh colour around stone was located on Chr4 and at three minor loci (Chr3, Chr6 and Chr8). ZYT, ‘Zhenzhuzaoyoutao’, CN14, ‘Zhongyoutao14’. RAS, red flesh around stone, WAS, white flesh around stone. (b), The major QTL locus for fruit maturity date was located at Chr4. HSM, ‘Huangshuimi’ (early maturing), ripening date about 85 days after flowering (DAF). QMH, ‘Qiumihong’ (late maturing), ripening date about 155 DAF. (c), The locus for double flowers was located at Chr2 and Chr6. HSM (single flower), SHLZ (‘Sahonglongzhu’, double flower).

Anthocyanins have crucial biological functions and affect quality of horticultural produce. Red colour around stone is essential for fruit coloration. Using GWAS, a previous study identified a candidate gene on Chr3 that encoded a *PpMYB10* transcription factor (Yamamoto *et al*., 2005). Earlier studies found three SNPs (Scaffold 6: 2,183,867, Scaffold 8: 16,905,885 and Scaffold 8: 16,795,565) that were strongly associated with red flesh using GWAS (Cao *et al*., 2016). In this study, the SNP most strongly related to red flesh was located on Chr4, while three association peaks were also found on Chr3, 6 and 8 (Table 2 and Figure 7a). The locus on Chr4: 10,514,015–11,601,515 bp included 143 genes. Among these genes that are predicted to encode two UDP‐glycosyltransferase superfamily proteins (Pp04G016610 and Pp04G016630) and six transcription factors (two NAC Pp04G017810 and Pp04G017800, two homeobox Pp04G016540 and Pp04G016670, one bZIP Pp04g016470 and one AP2/EREBP Pp04G016810). These genes might contribute to anthocyanin accumulation. There were three additional weak association peaks at Chr8: 19,929,060 bp, Chr3: 18,905,188 bp and Chr6: 13,088,613 bp. These loci included some genes that might contribute to anthocyanin accumulation, such as Pp08G024560 on Chr8 which encodes a MYB‐like transcription factor; Pp06G024190 on Chr6 which encodes a WRKY transcription factor; four MYB transcription factors on Chr3 (Pp03G021660, Pp03G021030, Pp03G021610 and Pp03G021670) and one bHLH transcription factors (Pp03G021150). These candidate genes require further investigation for their involvement in anthocyanin biosynthesis.

Fruit maturity date is an important trait for peach harvest and marketing. In previous study using GWAS, a locus for maturity date was located on Chr4 (Pirona *et al*., 2013). The candidate gene, a NAC transcription factor (Prupe.4G186800) containing a 9‐bp insertion in the coding region, showed a higher transcript level in an early harvest mutant compared to wild type (Pirona *et al*., 2013). In this study, the association peaks (Chr4: 11,550,220 bp) were near the gene of Pp04G017800 (Chr4: 11,558,954 bp, NAC transcription factor) (Table 2 and Figure 7b), which encodes the same gene as Prupe.4G186800. Interestingly, a three‐amino acid insertion in the third exon was found in Pp04G017800 (Figure S11). Within this locus, some other transcription factors were identified, such as two NAC (Pp04G016670 and Pp04G017810), two HB (Pp04G016540 and Pp04G016670) and one bZIP (Pp04G016470) transcription factors, as well as some hormone metabolism genes, such as the ABA‐related gene Pp04G017700, the IAA‐responsive gene Pp04G017680 and the ethylene‐related gene Pp04G017710. These candidate genes might contribute to fruit maturity date.

Double flowers are another important trait that is used in ornamental cultivars, especially in Asian countries. In this study, two significant peaks on Chr2 and Chr6 (Table 2 and Figure 7c) were associated with the double‐flower trait. In the locus on Chr2: 24,849,526–25,246,012 bp, the interval contained 64 genes, from Pp02G028200 to Pp02G0288300. The most promising candidate gene may be Pp02G0287000, which is similar to the homeotic gene *AP2*, a member of the TARGET OF EAT (TOE) genes and known to be involved in flower development. Interestingly, the gene Pp06G011800 (Prupe.6G242400) on Chr6, also encoding a TOE‐type transcription factor, was identified as a candidate gene for the *Di2* locus, another double‐flower trait that is inherited in a dominant manner (Gattolin *et al*., 2018). The interval region in Chr6: 23,153,445–23,907,278 bp was detected in this study, which was about 363.2 Kb to the Pp06G011800 gene.

Peach flesh colour ranges from white, yellow and red and is another important trait affecting fruit quality. A previous report indicated that the locus determining yellow/white colour was located on linkage group 1, that the carotenoid cleavage dioxygenase 4 (*CCD4*) gene is the most important gene for the yellow/white trait and that this gene was regulated by multiple modifier genes (Falchi *et al*., 2013). The yellow and white colour trait was analysed by GWAS for SNPs found in the 334 peach accessions (Table 2 and Figure S12a). The SNP most strongly related to yellow/white flesh was located on Chr1: 27,076,667 bp, about 171 Kb away from the *CCD4* gene (Pp01G032380, Chr1: 27,248,040 bp).

The peach kernel can be sweet or bitter, an important trait for processed kernel products. GWAS identified an association peak on Chr8 and 2 (Table 2 and Figure S12b). At Chr8: 3,272,636–4,136,977 bp, the highest associated peak was located in the promoter of Pp08G004100, which encodes a disease resistance protein. Two NAC transcription factors (Pp08G004140 and Pp08G004150) were also located in this area. On Chr2, three phenylpropanoid biosynthesis genes (Pp02G031770, Pp02G031580 and Pp02G032190) were located in this area, as were some transcription factors, such as AP2/EREBP (Pp02G031760 and Pp02G032140), bHLH (Pp02G031730), MYB (Pp02G031740) and MYB‐like (Pp02G031850). These genes might contribute to a bitter taste in the kernel.

For the pollen fertility trait, the dominant locus was located on Chr6, in the 1,708,346 to 2,288,022 bp interval (Table 2 and Figure S12c). A total of 93 genes are predicted in this 500‐Kb region. According to functional annotation, there were some genes that might be related to pollen fertility, such as Pp06G039250, an ATP synthesis‐related gene, Pp06G038900, an ATP‐binding cassette transporter G26 (ABCG26) involved in tapetal cell and pollen development, and two tetratricopeptide repeat (TPR)‐like superfamily genes (Pp06G038980 and Pp06G039150).

Flower bloom date, full bloom date and bloom ending date are important traits for ornamental cultivars. These three traits all mapped to Chr1: 26,970,777–27,985,365 bp (Table 2 and Figure S13a,b and c), where the association peak was located at the promoter and CDS region of Pp01G032380, which encoded the abscisic acid (ABA) synthesis gene 9‐*cis*‐epoxycarotenoid dioxygenase 4 (*NCED4*). *NCED4* might be a candidate gene for controlling flower bloom date. In this locus, we also identified two MYB transcription factors (Pp01G032100 and Pp01G033110) and one WD40 transcription factor (Pp01G032170) that might also involve in flower bloom date.

**Figure 8:**
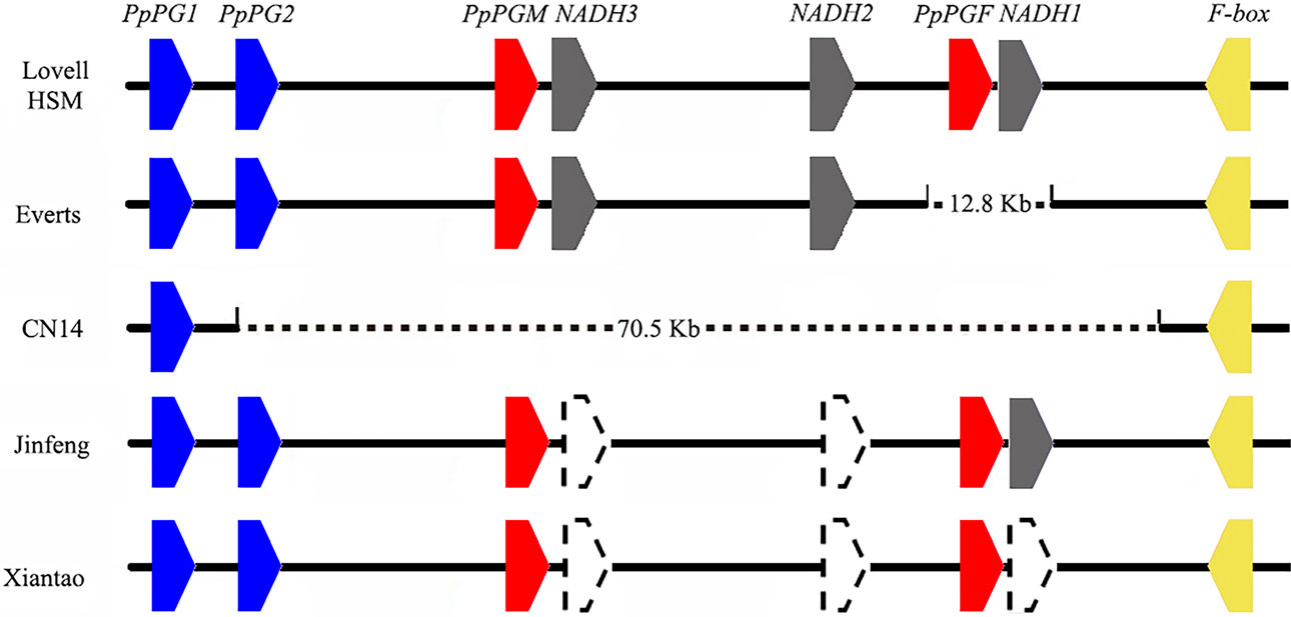
Five representative haplotypes of the *F‐M* locus in peach based on re‐sequencing data. *PpPGM*, the candidate gene for melting/non‐melting. *PpPGF*, the candidate gene for clingstone/freestone. Lovell and HSM represent haplotype with no deletion in *F‐M* locus, showing melting and freestone. Everts represent haplotypes with 12.8 Kb deletion, showing melting and clingstone. CN14 represents haplotypes with 70.5 Kb deletion, showing non‐melting and clingstone. Jinfeng and Xiantao represent haplotypes with two genes and three genes deletion respectively. Deletion is marked by dotted box.

The GWAS for flesh texture and flesh adhesion did not produce any significant association signal. A comparative genomics approach found a large gap of ~70.5 Kb in the *F‐M* locus in CN14. This suggested that CN14 was a clingstone non‐melting flesh. This might be the cause of the failed association study. The genotypes of the *F‐M* locus were analysed in the 344 peach accessions. There were five allelic variants, including no deletion in HSM (consistent with Lovell genome v2.0); a 12.8‐Kb deletion (contains *PpPGF* and *PpNADH1*) in Everts; three genes deletion (*PpNADH1*, *PpNADH2* and *PpNADH3*) in Xiantao; two genes deletion (*PpNADH2* and *PpNADH3*) in Jinfeng and a 70.5‐Kb deletion in CN14 covering *PpendoPGF*, *PpNADH1*, *PpNADH2*, *PpNADH3*, *PpPGM* and *PpPG2* (Figure 8 and Figure S14).

## Discussion

*De novo* assembly of a genome has proven to be beneficial to understanding various traits in peach. In this study, a high‐quality peach genome was constructed from a semi‐dwarf peach variety. The CN14 genome showed very high collinearity to the Lovell genome (v2.0), but the sequence anchored to chromosome size, contig N50 size and LAI value were all longer or higher than the Lovell genome (v2.0), while the gaps were shorter. These results indicated that the CN14 genome was at a high level of quality. Compared with the Lovell genome (v2.0), there were 75210 SVs. While most of the SVs were small (1–10 bp), there was a major difference on Chr3 between the two genomes, including 97 large SVs (inversions) according to collinearity and Assemblytics analysis. To understand the large‐scale difference on Chr3, four peach genomes (Lovell v2.0, RYP1, 124Pan and LHSM) were compared with CN14. CN14 was showing a similar variations between Lovell v2.0 and 124Pan, a large inversion with RYP1, and a high collinearity with LHSM (Figure 1c). To further identify these large SVs on Chr3, 20 large SVs were randomly selected for analysis using PCR, and most of these SVs were found in CN14 and the five other peach accessions (Figure 1e). The PCR results, high genome and gene BUSCO values and the ‘gold’ level LAI value all indicated that the CN14 genome was a complete and correct version.

Numerous genes were found to be expanded or contracted in the CN14 genome, including genes involved in secondary metabolism. There were 21 isoprenoid‐related genes that were in the decreased orthogroups; the reduced expression of these genes might result in lower aroma in the peach fruit. Interestingly, 21 sugar transport genes were in the expanded orthogroups, and 18 acid transport‐related genes, such as proton pump (P‐ and V‐ATPase) genes, were in the decreased orthogroups. The expansion/contraction of sugar and acid transport genes might contribute to sweet/acid taste of the peach fruit. Some genes related to phenylpropanoid and isoprenoid biosynthesis were found in expanded/decreased orthogroups and were related to fruit texture and aroma respectively.

AFDDD mapping, QTL/MTL mapping and GWAS approaches were used to identify genes controlling *TSSD*, flower type and flower size based on the whole‐genome re‐sequencing approach. AFDDD mapping is an effective approach for QTL/MTL discovery in out‐crossing woody species, using <lm×ll> and <hk×hk> type variants (Dougherty *et al*., 2018). The software GACD has been applied to construct a genetic map and for QTL/MTL mapping of an F_1_ population (Zhang *et al*., 2015). GWAS has successfully provided valuable genetic information and determined candidate genes controlling major agronomic traits in many species, such as *OsSPL13* controlling grain size in rice (Si *et al*., 2016) and *PpOFP* controlling peach fruit shape (Guan *et al*., 2021; Guo *et al*., 2020; Zhou *et al*., 2019). QTL/MTL and GWAS, each having their own merits and demerits, were recently combined to uncover loci governing agronomic traits (Sonah *et al*., 2015). In this study, loci found by QTL/MTL mapping of *TSSD*, flower type and size traits were validated by GWAS. For *TSSD*, a locus on Chr3 was detected within a 2‐Mb region by AFDDD and within a 0.24‐Mb region (4.74–4.98 Mb) by QTL/MTL. GWAS detected a leading SNP at base pair 4,376,637 bp of Chr3 that was highly associated with *TSSD*. The previous report mapped the locus to an interval of 500 Kb in scaffold 3 (Lu *et al*., 2016). Combined with transcriptome analysis, *PpTIP2*, encoding an aquaporin protein, was located in the QTL/MTL region and showed different expression profiles between CN14 (*TSSD*) and HSM (*tssd*). Recent studies showed that aquaporins are affected by multiple factors, such as hormonal signals, temperature or light (Maurel *et al*., 2015). In rice, five aquaporins were markedly up‐regulated during rapid internode elongation (Muto *et al*., 2011). In *Arabidopsis thaliana*, AtTIP5;1 promoted a hypocotyl elongation response to GAs (Pang *et al*., 2018). The promoter from CN14 (*TSSD*) showed different capacities in regulating GUS gene expression at different temperatures, with the GUS expression higher at 32 °C than that at 26 °C. A previous study showed that genes involved in signalling pathways of hormones, such as auxin, abscisic acid and jasmonic acid, were up‐regulated in response to elevated temperature in CN14, while genes related to cell expansion, such as expansin, polygalacturonase and endoglucanases, showed the same expression patterns (Lian *et al*., 2020). Expansin genes are up‐regulated by hormones to promote cell wall loosening and hypocotyl elongation (Quint *et al*., 2016). We speculated that high temperature induced hormone biosynthesis and signalling, then promoted the expansion‐related genes expression, such as *PpTIP2*, resulting in cell elongation. Therefore, the variations in the promoter of *PpTIP2* would result in different expression levels of *PpTIP2* at different temperatures to confer the temperature‐sensitive semi‐dwarf phenotype in CN14. Our knowledge about the functional links among the components of the temperature perception and signalling network remains limited (Casal and Balasubramanian, 2019). While the need to explore the genetic variability of temperature perception and signalling in organisms other than *Arabidopsis thaliana* is necessary for our response to a changing climate (Casal and Balasubramanian, 2019). These results will facilitate developing markers for *TSSD* selection and enriching our understanding of the regulatory network of temperature sensing.

The inheritance of flower type in peach was found to be determined by a major gene (*SH/sh*,) with non‐showy flower dominant over showy flower (Bailey and French, 1942). Previous research mapped the *SH* gene to LG8 (Cao *et al*., 2016; Fan *et al*., 2010). A clear association signal was located at 13,740,117 bp on scaffold 8 in the up‐stream region of the gene ppa016980m (Cao *et al*., 2016). In this study, flower type was highly associated with a SNP detected in the promoter region of the *PpB3‐1* gene located on Chr8 by GWAS. The SNP was also located in this region by AFDDD and QTL/MTL. Meanwhile, the same locus was identified by GWAS using 344 peach accessions (Figure 4a). The SNP was confirmed to be associated with flower type trait in 88 peach cultivars and 73 hybrids through sequencing. Additionally, *PpB3‐1* showed different expression profiles between showy and non‐showy flower petals. The B3‐type transcription factor superfamily has been suggested to be involved in growth and development of aerial lateral organs and to negatively regulate cell proliferation of lateral organs (Lee *et al*., 2015). It has been reported that overexpression of *AtNGA1* (NGATHA) to *AtNGA4* led to small, narrow petals, whereas the opposite phenotype was observed in the *AtNGA_1,2,3,4_* quadruple mutant (Lee *et al*., 2015). The expression level of *PpB3‐1* was higher in non‐showy flowers (small petal) than in showy flowers (*sh*) in this study. Taken together, *PpB3‐1* may negatively regulate petal growth in peach.

Flower size is controlled by different pairs of genes (Bailey and French, 1942). Based on the phenotypic data of flower size over 3 years, loci related to flower size were identified at the same locus to that of flower type in this study. This was the first report of mapping for flower size and suggested that the major QTL for flower size may be located at the same loci of *SH*.

The high‐quality genome supplied a powerful foundation for identifying the genetic variations underlying agronomic traits. Although GWAS for agronomic traits has been performed in several studies, all were based on the Lovell v2.0 genome. In this study, we identified more precise association signals than previously identified based on the high‐quality CN14 genome. For instance, two significant peaks on Chr2 and 6 were associated with double flowers in this study. There are two distinct loci controlling the double‐flower phenotype in peach. One was assigned to linkage group 2 (Cao *et al*., 2016; Dirlewanger *et al*., 2004), and the other was mapped to Chr6 (Pascal *et al*., 2017), while in this study, the two loci were identified using GWAS analysis. The present results identified Pp06G011800, located on Chr6, as the candidate gene for the double‐flower trait. Notably, Pp06G011800 encodes a TARGET OF EAT (TOE)‐type euAP2 transcription factor and was annotated as Prupe.6G242400 in Lovell genome v2.0, which was also named a candidate gene for the double‐flower trait (Gattolin *et al*., 2018). Meanwhile, the TOE‐type AP2 gene Pp02G0287000 on Chr2 may be another candidate gene for the double‐flower trait. For the other agronomic traits mentioned in this study, candidate genes or loci were identified in previous studies (Guo *et al*., 2020), and we also identified these loci using GWAS with the CN14 genome (Figure 6, 7; Figure S12, Figure S13). A TE insertion in the gene Prupe.5G196100 was reported to be associated with the fruit hairless trait (Vendramin *et al*., 2014), and herein a 6015‐bp insertion was identified in the third exon of Pp05G004700 (the same gene as Prupe.5G196100) (Figure 6, Figure S10). These results further clarify that the variation of *PpMYB25* results in the peach and nectarine phenotypes. In previous study, the yellow flesh trait was located on scaffold 1: 24,968,892 bp, about 0.67 Mb away from *PpCCD4* (Cao *et al*., 2016), while in this study, the SNP was located on Chr1: 27,248,040, about 171 Kb away from Pp01G032380 (*PpCCD4*). For red flesh around stone, a 487‐bp deletion in the promoter of Prupe.3G163100 (*PpMYB10.1*) was highly associated with the phenotype (Guo *et al*., 2020; Zhao *et al*., 2020). In this study, some candidate genes in three new loci might contribute to red flesh phenotype. For flower bloom date, a locus was found on Chr1: 26,970,777‐27,985,365 bp, with no obvious signals, in a previous study (Guo *et al*., 2020), while here the candidate SNP was located to the promoter and CDS of Pp01G032380 (NCED4), indicating that ABA might be involved in flower bloom date. In two previous studies, fruit maturity date was reported to be associated with changes in the CDS of Prupe.4G186800 (Guo *et al*., 2020; Pirona *et al*., 2013), and here we identified a three‐amino acid insertion encoded in the third exon of Pp04G017800. Previous studies indicated that the three amino acids contributed to maturity date, and CN14, an early ripening cultivar, carries the three‐amino acid insertion, further confirming that maturity date is located on Chr4 and that Pp04G017800 is a candidate gene for early fruit maturity.

Fruit texture is an important attribute affecting consumer perception of fruit quality. Two new variants were detected in the *F‐M* locus in this study, in addition to three variants reported in a previous study (Gu *et al*., 2016). Peach melting flesh and flesh adhesion to stone (endocarp) are simply inherited and controlled by the *F‐M* locus on linkage group 4 (Ogundiwin *et al*., 2009). Within the *F‐M* locus, *PpendoPGF* and *PpendoPGM*, two genes encoding endopolygalacturonase (*endoPG*), were associated with the stone adhesion and melting flesh traits respectively (Gu *et al*., 2016). The *F‐M* locus has three allelic copy number variants, including H1 with no deletion, H2 with a 12.8‐Kb gap and H3 with a 70.5‐Kb gap (Gu *et al*., 2016). In addition, two new variants (three genes deletion and two genes deletion) were also detected in *F‐M* locus in this study. A previous study found that it was easy to identify flesh texture and stone adhesion by detecting the presence or absence of *PpendoPGM* and *PpendoPGF* respectively (Gu *et al*., 2016). On the other hand, it was difficult to identify homozygous or heterozygous freestone melting flesh (FMF) or clingstone melting flesh (CMF). The results here will lay a foundation for further dissection of identification of FMF and CMF based on the five allelic variants.

## Conclusion

A high‐quality peach genome was generated from a cultivar with a promising agronomic phenotype. Comparison of this genome with the earlier versions developed from a peach rootstock has deepened our population‐level understanding of genomic variation. The major gene for the temperature‐sensitive semi‐dwarf trait (*TSSD*) was mapped to Chr3 and found to be *PpTIP2*. The major gene for flower type (*SH*/*sh*) and flower size was mapped to Chr8 and identified as *PpB3‐1*. GWAS was used to identify candidate genes that were highly associated with double flowers, fruit flesh colour (yellow/white), flesh colour around the stone, pollen fertility, kernel taste, flower bloom date, fruit maturity date and hairiness/hairless variant. The variation of a 6015‐bp insertion in the third exon and a 26‐bp insertion upstream of *PpMYB25* were associated with fruit hairiness/hairless. Two novel allelic variants were identified in the *F‐M* locus. This study lays the foundation for the further understanding of the regulatory mechanisms of shoot elongation at elevated temperatures and other agronomic traits. The genetic resources identified in this genome can be used for peach molecular‐assisted breeding.

## Materials and methods

### Plant materials and sampling

The cultivar CN14 (*TSSD*, *SH*, small flower and hairless) was used to construct a high‐quality genome. Young leaves of CN14 were used for DNA extraction *via* Dzup Genomic DNA isolation reagent (Sangon Biotech Co.). Fruits at four stages (SI, SII, SIII and SIV stages as defined by previous study) (Tonutti *et al*., 1997), young leaves, mature leaves, shoot tip and stem of CN14 were used for RNA extraction *via* Plant Total RNA Isolation kit (B518631, Sangon, Shanghai, China), these RNA were used for transcriptome sequencing and contributing to protein‐coding gene identification. The cultivar CN14, ‘Huangshuimi’ (HSM), ‘Okubo’, ‘Matsumori’, ‘Fenshouxing’ and ‘Phillips’ were used for verification of the large inversions on Chr3.

An F_1_ population of 86 individuals was generated from a cross between HSM (female parent, *tssd*, *sh*, large flower and hairiness) and CN14 (male parent). This population was used to identify candidate genes for the *TSSD*, *SH* and flower size traits. Young leaves were harvested from the two parent plants and each individual progeny plant for DNA extraction. The DNA of these 88 samples was sequenced by Illumina HiSeq^TM^ 2500 platform by PE125 mode.

All peach materials used in this study were maintained in the field under normal cultural conditions at the Henan Agricultural University peach repository, Zhengzhou, China.

Based on the growth habit of *TSSD*, terminal internode length of CN14 and HSM was measured at four critical stages (initial period, IP; initial elongation period, IEP; rapid growth period, RGP; and stable growth period, SGP) (Lian *et al*., 2020). Three biological replicates were collected.

Flower development was classified into five periods, namely BP (buds period), RDP (red dot period), EP (equivalent in size between petal and sepal period), BFP (budding flower period) and FBP (full bloom period). RNA was extracted from the petals of CN14 and HSM at the five periods used for RNA‐seq according to Plant Total RNA Isolation kit (B518631, Sangon, Shanghai, China).

### Paraffin sectioning

The annual stem base (~1 cm) from CN14 and HSM was fixed and sectioned according to previously reported method (Cheng *et al*., 2019). An optical microscope (Nikon eclipse E100) was used for visual analysis.

### Sequencing and assembly of Genomic DNA from CN14

Genomic DNA of CN14 was extracted from 0.1 g of leaf sample using the Dzup Genomic DNA isolation reagent (Sangon Biotech Co.) according to the protocol. The quality of the DNA was assessed using gel electrophoresis and NanoDrop 2000 spectrophotometer (Thermo Scientific).

Genomic DNA was used to construct paired‐end libraries following the manufacturer’s instructions for high‐throughput DNA sequencing. A 300–400 bp overlap library was constructed. The final library was sequenced using BGISEQ platform by paired‐end 150‐bp mode. The raw data were filtered to remove low‐quality reads and adaptor sequences using SOAPnuke software (v1.5.6) with the following parameters (‐n 0.01 ‐l 20 ‐q 0.1 ‐i ‐Q 2 ‐G ‐M 2 ‐A 0.5 ‐d).

For the SMRTbell libraries, the DNA was fragmented into 20‐kb segments that were used to construct a 20‐Kb library using the SMRTbell Template Prep kit (Sage Scientific, MA). SMRT cells were loaded and run on the Sequel II system at BGI (Shenzhen, China).

The Pacbio long reads were used to assemble the genome using CANU (v1.9). Firstly, reads were corrected based on overlap sequences, then low‐quality reads were trimmed and finally the corrected reads were used to assemble the genome. To improve the accuracy of the genome, Pacbio long reads were used to correct the contigs using Racon (v1.4.3) three times, and then the short reads (PE150 mode) were mapped to the peach genome. These short reads were then used to correct the contigs using Pilon (v1.23).

To better build the genome to the chromosomal level, a Hi‐C library was constructed according to a published protocol (Rao *et al*., 2014). In brief, 2 g of young leaves were cross‐linked *in situ* using 1% formaldehyde solution. Chromatin was extracted and digested using *Mbo*I (New England Biolabs), and then the DNA was labelled, biotinylated, diluted and randomly ligated. The DNA fragments were enriched and quality checked, then the sequencing libraries were constructed and sequenced using BGISEQ‐500 platform by 100‐bp paired‐end mode. Hi‐C sequenced data were filtered using HicPro (v2.11.1), then the clean data were aligned to the corrected contig genome using Juicer (v1.6). The mapped data were used to construct a draft assembly genome using 3D‐DNA (v180922), and the draft genome was corrected using Juicerbox. According to the correlation between the different contigs, the genome was adjusted using HicPlotter (v0.6.2), and finally a pseudo‐molecule chromosome genome was obtained using D‐GENIE according to the reference genome (Lovell v2.0).

Two methods were used to assess the accuracy of the assembled genome and to evaluate the quality of the genome. First, the eukaryota_odb9 database, including 1440 genes, was used to measure the completeness of the genome using benchmarking universal single‐copy orthologs (BUSCO v2.0) (Manni *et al*., 2021). Secondly, the LTR Assembly Index (LAI) was used to evaluate genome completeness (Ou *et al*., 2018).

### Genome annotation

For annotation of repeat sequences, mainly including tandem repeat and interspersed repeats, two comparisons were made, homolog and *de novo*. First, the RepBase database (v21.12) was used to identify repeats using RepeatMasker (v4.0.7) and RepeatProteinMask (v4.0.7). Second, Piler (v0.1.23), RepeatScout (v1.0.5) and RepeatModeler (v4.0.7) were used to build a *de novo* repeat database, and Repeatmasker was used to identify repeat sequences.

Gene models were identified according to homology, *de novo* and RNA‐Seq‐based predictions. The proteins and mRNA sequences from eight species (*Fragaria vesca*, *Rosa chinensis*, *Prunus dulcis*, *Malus* × *domestica*, *Pyrus ussuriensis* × *Pyrus communis*, *Malus caccata*, *Prunus mume* and *Prunus avium*) were subjected to BLAST searches with the CN14 genome. Matches with scores > 90% were subjected to Exonerate (v2.2.0) and Genewise (v2.4.1) to refine the gene models of the CN14 genome. For *de novo* predication, Augustus (v3.4.0) and GlimmerHMM (v3.0.4) software were used. For RNA‐Seq‐based prediction, transcriptome sequences from a mixed sample (fruit at SI, SII, SIII and SIV stages, young leaves, mature leaves, shoot tip and stem) were used. RNA‐Seq data were mapped to the CN14 genome using hisat2. The transcripts were obtained by Cufflinks (v2.2.1). Finally, EVM (v1.1.1) was used to merge all gene models from previous methods to generate the final consensus gene model of the CN14 peach genome. To understand the accuracy of the gene models, BUSCO was used to measure gene model completeness, using the Insecta_odb10 database (1361 genes) as a query.

The potential functions of genes were annotated based on eight protein databases, including COG (https://www.ncbi.nlm.nih.gov/COG/), GO (http://geneontology.org/), eggNog (http://eggnog5.embl.de/app/home#/app/home), KEGG (https://www.kegg.jp/), NR (https://www.ncbi.nlm.nih.gov/), SwissPort (https://www.uniprot.org/downloads), Rice (http://rice.uga.edu/) and Tair (https://www.arabidopsis.org/) databases. Functions were annotated when they matched with an e‐value of 1e^‐10^.

### Gene family expansion and contraction analysis

Gene family expansion and contraction were analysed by comparing cluster size differences between the CN14 peach, Lovell peach genome (v2.0) and 12 other plant genomes (*Prunus mume*, *Prunus apricot*, *Prunus salicina*, *Prunus dulcis*, *Prunus communis*, *Malus* × *domestica*, *Rose chinensis*, *Carica papaya*, *Arabidopsis thaliana*, *Populus trichocarpa*, *Vitis vinifera* and *Oryza sativa*) using the CAFE program (v4.2). The functions of expanded and contracted genes were annotated using Mercator (v3.6, https://www.plabipd.de/).

### Structure variation analysis between CN14 and Lovell v2.0 genome

To identify SVs between the CN14 and Lovell v2.0 genomes, the two genomes were analysed using MUMmer (v3.0). The SVs were called using Assemblytics (http://qb.cshl.edu/assemblytics/). To compare the large SVs on Chr3, CN14 was compared with Lovell (v2.0), 124Pan (Zhang *et al*., 2021), RYP1 (Guan *et al*., 2021) and LHSM (Yu *et al*., 2021) genomes by MUMmer, and then mapped using gnuplot (v5.2.8). To further study the variation in the SVs on Chr3, 20 SVs were randomly selected to design primers using Primer Premier 5 (Table S14). The SVs were amplified by PCR in CN14 and five other peach germplasms.

### Phenotype of HSM, CN14 and their F_1_ population

Three traits, including growth habit (*TSSD*/*tssd*), flower type (*SH*/*sh*) and flower size, were investigated in the F_1_ population (HSM × CN14). Based on the growth habit of *TSSD*, terminal internode length of CN14 and HSM was measured at four critical stages (IP, IEP, RGP and SGP) (Lian *et al*., 2020).

Flower type was classified according to the petal size and shape. Showy flowers were large with a broad petal, while non‐showy flowers were small with a narrow petal. Flower size was calculated as the diameter of a flower at full bloom. Ten flowers were measured for each progeny, and phenotypic evaluation was conducted for three consecutive years (2018, 2019 and 2020). The data were analysed using SPSS version 17.0 (SPSS, Chicago, IL).

For GWAS, the phenotypes, including flesh colour (yellow/white and flesh colour around stone), hairiness/hairless, fruit maturity date, flower size, double flowers, flower opening date (flower bloom date, full bloom date and bloom ending date), pollen fertility, flesh texture, flesh adhesion and kernel taste, of 334 peach accessions were according to a previous report (Guo *et al*., 2020).

### Genome re‐sequencing and GWAS analysis

Genomic DNA from CN14, HSM and their F_1_ progeny was used to construct 350 to 400 bp libraries, which were then re‐sequenced using Illumina HiSeq2500 by paired end in 125‐bp mode. Raw data from the 334 peach accessions (PRJNA630113) were downloaded from the NCBI database (https://www.ncbi.nlm.nih.gov). All raw data were filtered to remove the adapters and low‐quality reads using Trimmomatic‐0.38. Cleaned reads were mapped to the reference peach genome (CN14) using BWA (v0.7.17‐r1188). The SAM files were converted into BAM file using SAMtools; BAM file was sorted using SAMtools mpileup. SNPs were identified using SAMtools (mpileup) and GATK (v4.1.9.0). The SNPs of all samples were merged using BCFtools (v1.7), then the SNPs were filtered using VCFtools (v0.1.15) and PLINK (v1.90b6.21). Finally, 496,610 SNPs were used for the GWAS in hybrid populations (HSM, CN14 and 86 individuals) and 846,176 SNPs were used for GWAS of the 334 natural populations.

SNPs were filtered based on allele frequency values lower than 0.1 and deficiency rates above 0.1 in all germplasms. In the TASSEL (v5.2.69) software, the GLM‐no PCA model was used for genome association analysis based on 88 and 334 peach re‐sequencing datasets respectively. Linkage disequilibrium (LD) was analysed used PLINK, and population structure was evaluated using admixture (v1.3.0), with the number of populations (K value) ranging from 2 to 10. The significance cut‐off was according to the Bonferroni test threshold, which was set as 0.05/(total number of SNPs), which served as 7.23 for natural populations and 7.00 for hybrid populations. Manhattan plot and QQ plot for each trait were drawn using R package qqman (v0.1.8).

### Linkage map construction

Raw genotypes of the SNPs were assigned according to the deduced parental genotypes. F_1_ individuals (HSM × CN14) were genotyped using the genotype SNPs by a hidden Markov model according to Xie *et al*. (2010). HighMap (Liu *et al*., 2014) and GACD (Zhang *et al*., 2015) programs were used to construct a genetic map based on the genotyped SNPs of F_1_ population (HSM × CN14). The MTL or QTL were used to identify candidate locus by the linkage map and phenotypic data. The LOD values were more than 2.0. The QTLs responsible for more than 10% of the phenotypic variation were considered major QTLs. The candidate genes were annotated by GO, KEGG and Nr databases.

### RNA sequencing and qRT‐PCR analysis

Total RNA was extracted using the Total RNA Rapid Extraction kit (Sangon, Shanghai, China). For each sample, 2 μg of high‐quality RNA was used for library construction and sequencing. The mRNA was purified from the total RNA, and then assessed using an Agilent Technologies 2100 Bioanalyzer (Agilent, United States). According to the RNA Preparation Guide, first‐ and second‐strand complementary DNA was synthesized. The double‐strand cDNA was purified, and adapters were added. The constructed RNA libraries were sequenced on the BGI‐SEQ‐500 platform in paired‐end 100 bp mode.

The low‐quality reads and adapters of the RNA‐Seq raw data were removed for all samples using Trimmomatic‐0.38. Clean reads were mapped to the CN14 reference genome using hisat2 (v2.1), transcript abundance was estimated using HTSeq (v0.12.3) based on the alignments. Differentially expressed genes (DEGs) were identified using the R package edgeR (v3.34.0). Gene expression patterns were grouped into nine clusters using the Mfuzz (v2.52.0) package in R. The heatmap of candidate genes was based on expression levels and analysed using TBtools (Chen *et al*., 2020).

To validate the results of RNA sequencing, the expression level of differentially expressed genes was detected using qRT‐PCR. The RNA was used to transcribe cDNA with PrimeScript RT kit (TaKaRa), then the cDNA was subjected to qRT‐PCR using a StepOnePlus Real‐Time PCR System (Applied Biosystems, Foster, CA). *PpRPL13* was the endogenous control gene. The primer sequences are listed in Table S14. The relative expression levels of genes were calculated using the 2^‐ΔΔCt^ method (Schmittgen and Livak, 2008).

### Promoter activity analysis using GUS assay in *N. benthamiana* leaves

Promoter regions 1500 bp upstream of *PpTIP2* in HSM and CN14 were cloned into the pCAMBIA1381‐GUS vector to generate the reporter constructs *PpTIP2‐HSM_pro_‐GUS* and *PpTIP2‐CN14_pro_‐GUS* respectively. The reporter constructs were transiently transformed into *N. benthamiana* leaves *via Agrobacterium‐*mediated infiltration (GV3101) as described for the promoter activity assay (Li *et al*., 2021). The plants were grown for 48 h at 26 °C and 32 °C in a growth chamber respectively. The empty pCAMBIA1381‐GUS vector (CK) was used as a negative control. GUS staining was performed according to the GUSblue kit manual (Huayueyang Biotechnology, Beijing, China).

### SNP validation of *PpTIP2* and *PpB3‐1* in peach accessions

The promoter region of *PpTIP2* was amplified in F_1_ hybrids from the cross HSM × CN14 (randomly selected 20 *TSSD* and 20 *tssd*) to validate co‐segregation of SNPs and *TSSD* phenotype. The candidate SNP in the promoter of *PpB3‐1* associated with *SH/sh* was amplified in 161 peach accessions (98 *SH* and 63 *sh*) with specific PCR primers (Table S14), including 88 natural accessions (55 *SH* and 33 *sh*) and 73 hybrids (43 *SH* and 30 *sh*).

The PCR products were sequenced by direct Sanger sequencing using the ABI3730xl DNA Analyzer (Applied Biosystems, Foster City, CA) at Sangon Company (Shanghai, China).

### Detection of DNA variants of *PpMYB25* for hairiness/hairless and *F‐M* locus in peach

To verify that the 6015‐bp inversion of *PpMYB25* was associated with hairiness/hairless, the DNA from 60 peach accessions (21 hairiness and 39 hairless) were sequenced by PCR using specific primers listed in Table S14. The PCR products were detected using agarose gel electrophoresis.

To detect the genomic structure variation in the *F‐M* locus, eight pairs of primers were designed for amplification whole sequence of *PpPG1*, *PpPG2*, *PpPGM*, *PpNADH3*, *PpNADH2*, *PpPGF*, *PpNADH1* and *F‐box* respectively. P38 and P39, designed in a previous study (Gu *et al*., 2016) were used to detect 12.8‐Kb gap and 70.5‐Kb gap respectively. All primer sequences were listed in Table S14.

## Conflicts of interest

The authors declare that they have no competing interests.

## Author contributions

J.F., X.L. and B.T. conceived the project. X.L. and H.Z. contributed equally to this work. H.Z., J.C., W.W. and X.Y. performed the sequencing. X.L., C.J., X.Z. and L.Y. conducted the data analyses. X.L., F.G., X.W. and J.L. carried out field experiment, phenotype investigation and sample collection. X.L. and L.Z. performed the RNA/DNA extraction. X.L., C.J. and Z.L. performed the experiment verification. J.F., X.L., H.Z. and B.T. designed the analysis and wrote the manuscript.

## Supporting information


**Figure S1** K‐mer frequency distribution and GenomeScope profile of the CN14 genome.
**Figure S2** Annotation of gene that showed expansion and contraction of gene numbers.
**Figure S3** Distribution of allele frequency of variants and significant variants (SVs) density peaks located on Chr3.
**Figure S4** MTL of temperature‐sensitive semi‐dwarf (*TSSD*) on the genetic linkage map.
**Figure S5** Relative gene expression of DEGs near the *TSSD* locus was analysed by qRT‐PCR in HSM and CN14 at four growth stages of terminal internode.
**Figure S6** Different *cis‐*elements in the promoters of the *PpTIP2* gene in CN14 and HSM.
**Figure S7** Distribution of density of variations associated with flower type across the eight peach chromosomes.
**Figure S8** MTL of flower type on the genetic linkage map.
**Figure S9** Relative gene expression of DEGs at the *SH* locus was analysed by qRT‐PCR in HSM and CN14 at five growth stages of flowers. The left *Y*‐axis indicates the relative gene expression; the right *Y*‐axis indicates the FPKM value.
**Figure S10** Identification of the 6015‐bp insertion in *PpMYB25* in 60 peach accessions.
**Figure S11** Sequence alignment of NAC6 in CN14 and Lovell genome.
**Figure S12** Identification of candidate genes for flesh colour (while/yellow), kernel taste and pollen fertility
**Figure S13** The same locus was associated with flower bloom date (a), full bloom date (b) and bloom ending date (c).
**Figure S14** Agarose gel electrophoresis of the PCR products in *F‐M* locus.

**Table S1** BUSCO statistics of genome and proteins.
**Table S2** Summary of transposable elements in CN14 genome.
**Table S3** Summary of gene annotation statistics.
**Table S4** Summary for structural variants between CN14 with Lovell v2.0 peach genome.
**Table S5** Summary of SVs in CN14 and Lovell v2.0 genome.
**Table S6** Five large inversions between CN14 and Lovell v2.0 genome.
**Table S7** List of genes involved in expansion and decrease.
**Table S8** Segregation analysis of tree habit and flower type in F_1_ progeny derived from HSM and CN14.
**Table S9** SNP variation of the promoters of CN14 and HSM.
**Table S10** Molecular identification of the variation in the promoter of *PpTIP2* in hybrids.
**Table S11** Molecular identification based on SNP in different flower type peach accessions.
**Table S12** A total of 344 natural populations were used in this study.
**Table S13** Molecular identification of the variation (a 6015‐bp insertion) in *PpMYB25* in natural populations with hairiness/hairless fruit types.
**Table S14** Primers used in this study.

## Data Availability

The genome assembly and annotation files are avaliable in the Peach Genome Database (http://www.stylebio.cn/index.html).
