# Supplementary material for: De novo chromosome‐level genome of a semi‐dwarf cultivar of Prunus persica identifies the aquaporin PpTIP2 as responsible for temperature‐sensitive semi‐dwarf trait and PpB3‐1 for flower type and size
Source: Plant Biotechnol J. 2022 Jan 4;20(5):886–902. doi: 10.1111/pbi.13767 (PMC9055816; doi:10.1111/pbi.13767)
Supplement: Supplementary file 1 — Figure S1 K‐mer frequency distribution and GenomeScope profile of the CN14 genome. Figure S2 Annotation of gene that showed expansion and contraction of gene numbers. Figure S3 Distribution of allele frequency of variants and significant variants (SVs) density peaks located on Chr3. Figure S4 MTL of temperature‐sensitive semi‐dwarf (TSSD) on the genetic linkage map. Figure S5 Relative gene expression of DEGs near the TSSD locus was analysed by qRT‐PCR in HSM and CN14 at four growth stages of terminal internode. Figure S6 Different cis‐elements in the promoters of the PpTIP2 gene in CN14 and HSM. Figure S7 Distribution of density of variations associated with flower type across the eight peach chromosomes. Figure S8 MTL of flower type on the genetic linkage map. Figure S9 Relative gene expression of DEGs at the SH locus was analysed by qRT‐PCR in HSM and CN14 at five growth stages of flowers. The left Y‐axis indicates the relative gene expression; the right Y‐axis indicates the FPKM value. Figure S10 Identification of the 6015‐bp insertion in PpMYB25 in 60 peach accessions. Figure S11 Sequence alignment of NAC6 in CN14 and Lovell genome. Figure S12 Identification of candidate genes for flesh colour (while/yellow), kernel taste and pollen fertility Figure S13 The same locus was associated with flower bloom date (a), full bloom date (b) and bloom ending date (c). Figure S14 Agarose gel electrophoresis of the PCR products in F‐M locus. [file PBI-20-886-s001.docx]

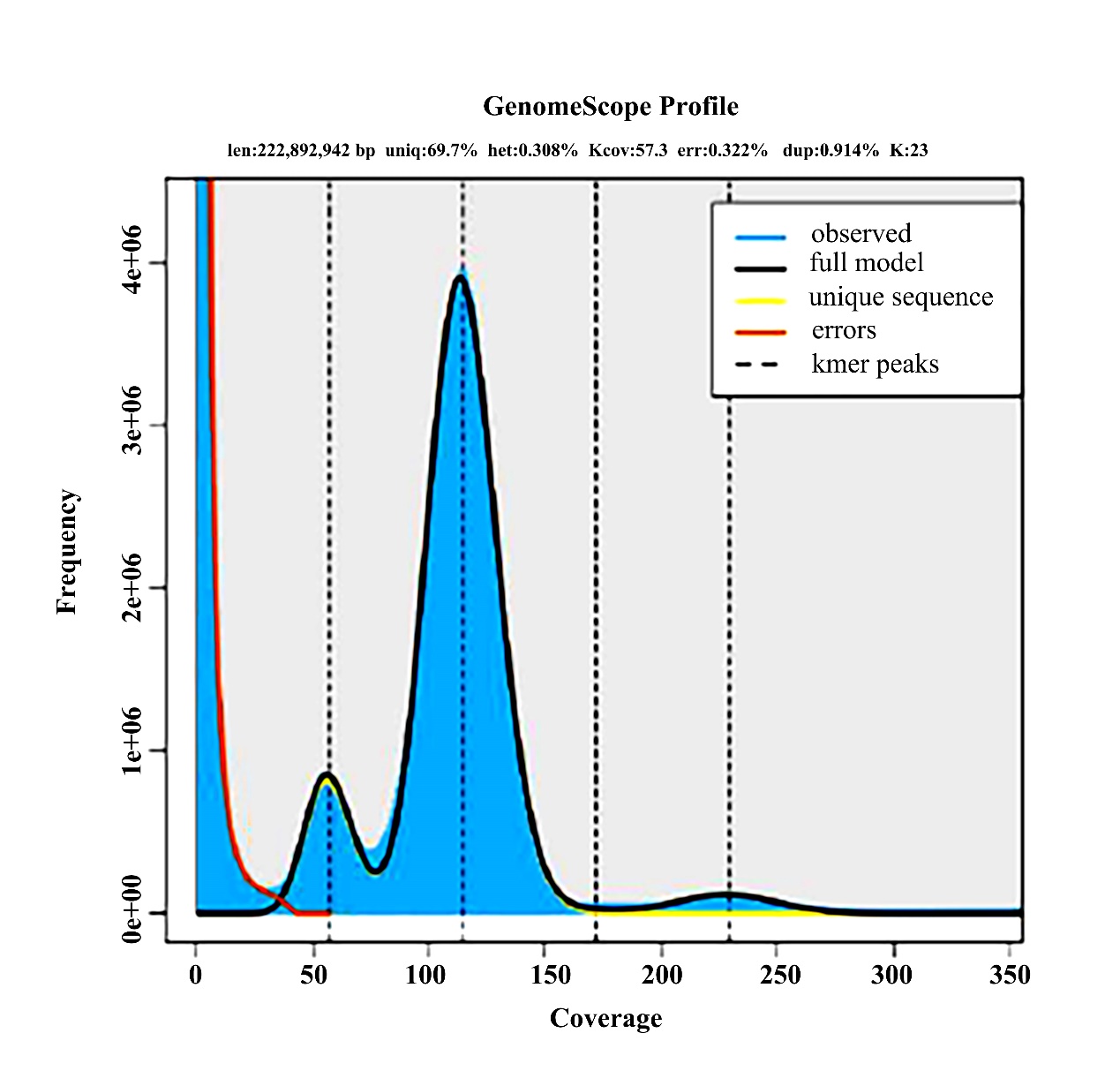


Figure S1 K-mer frequency distribution and GenomeScope profile of the CN14 genome. The estimated genome size was 222.89 Mb, with a heterozygosity rate of 0.31%.


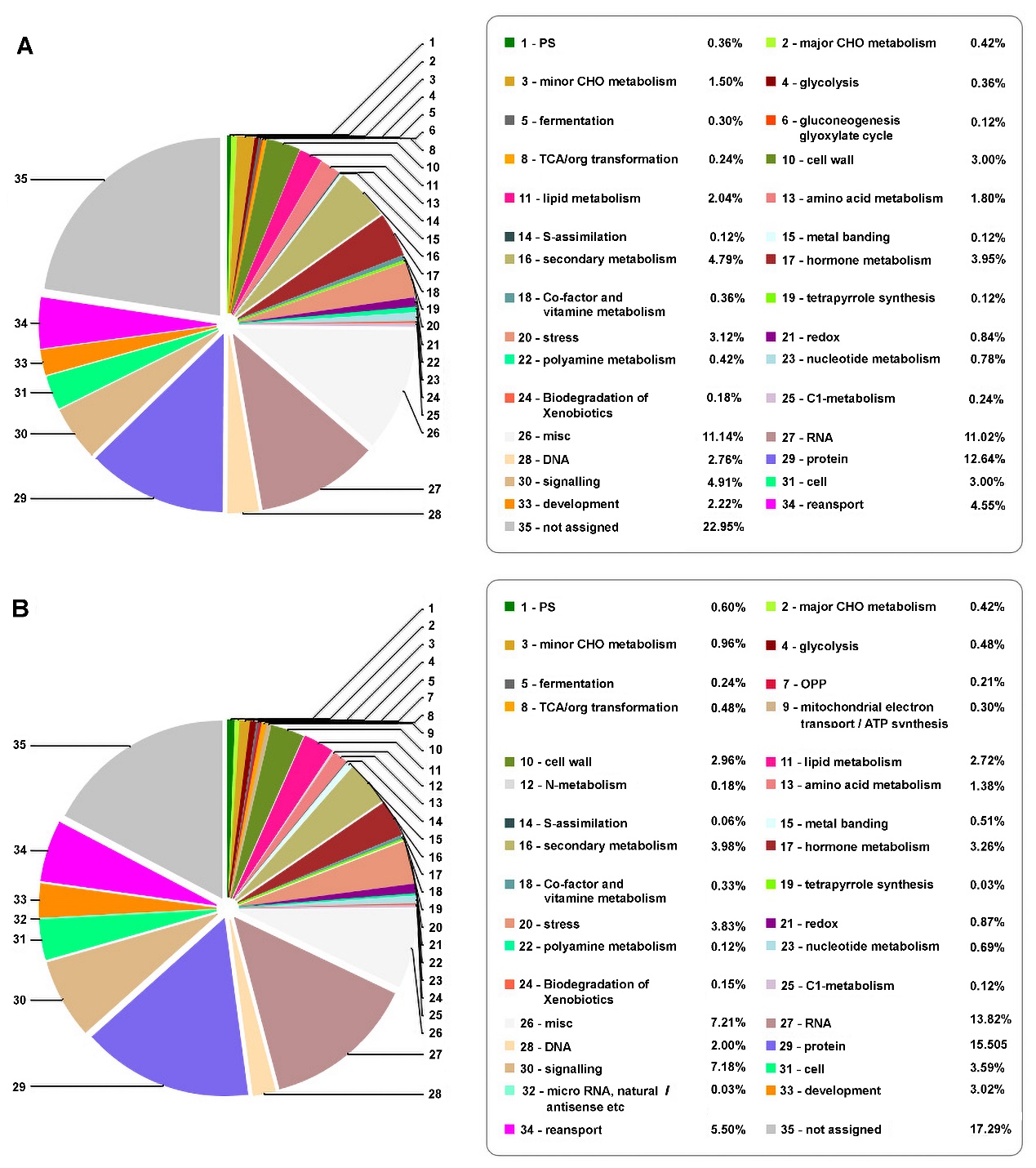


Figure S2 Annotation of gene that showed expansion and contraction of gene numbers. A, Functional annotation of genes in expansion orthogroups. B, Functional annotation of genes in contraction orthogroups.


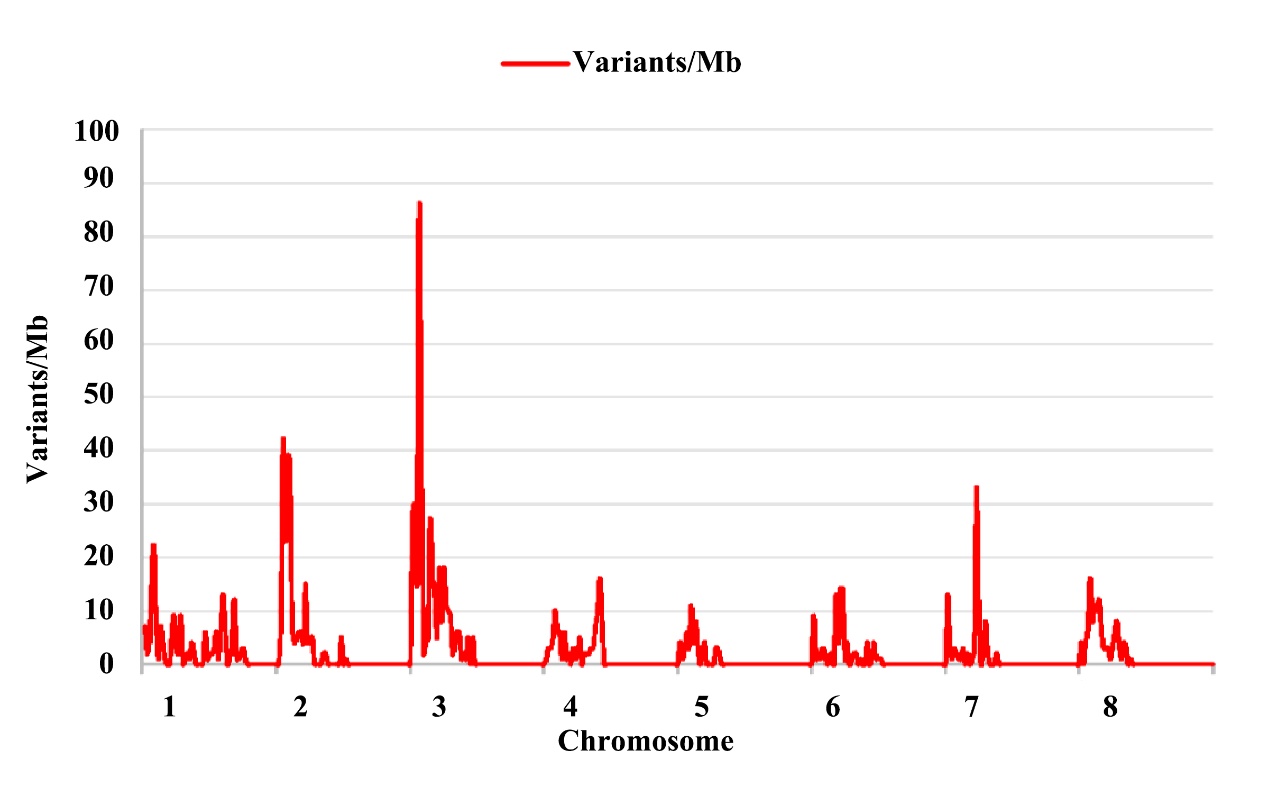


Figure S3 Distribution of allele frequency of variants and significant variants (SVs) density peaks located on Chr3.


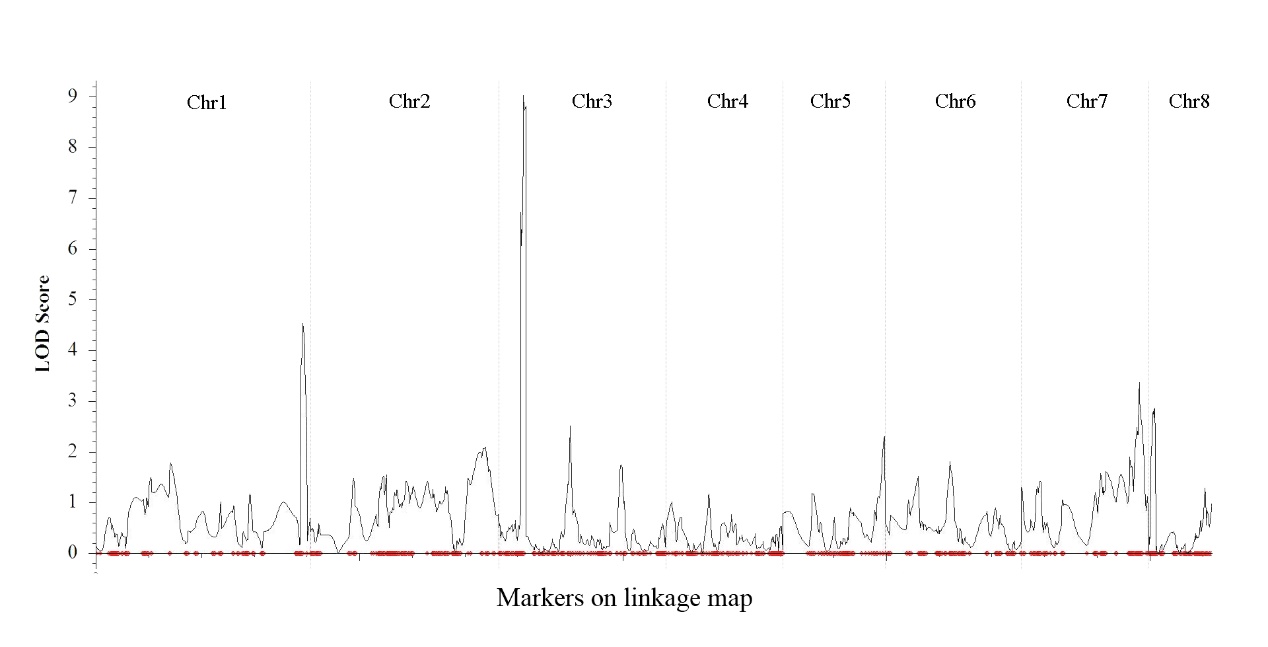


Figure S4 MTL of temperature-sensitive semi-dwarf (*TSSD*) on the genetic linkage map.


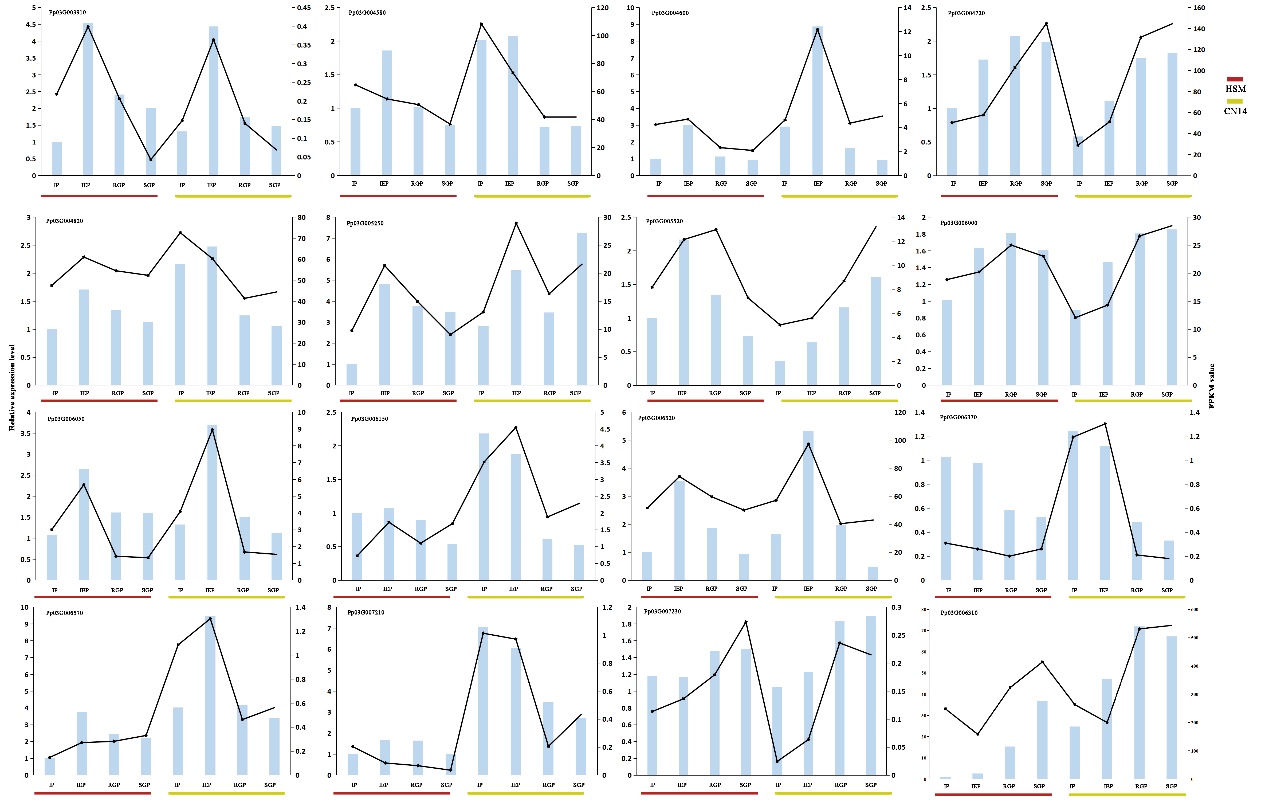


Figure S5 The relative gene expression of 15 DEGs in *TSSD* locus was analyzed by qRT-PCR in HSM and CN14 at four stages. The left y-axis indicates the relative gene expression; the right y-axis indicates the FPKM value.

**
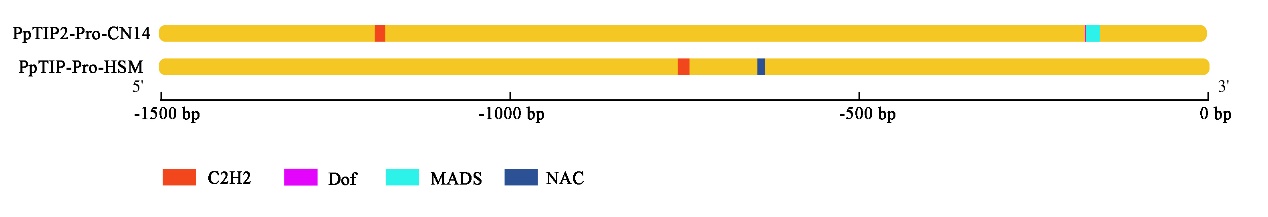
**

Figure S6 Different *Cis-*elements in the promoters of *PpTIP2* gene in CN14 and HSM.


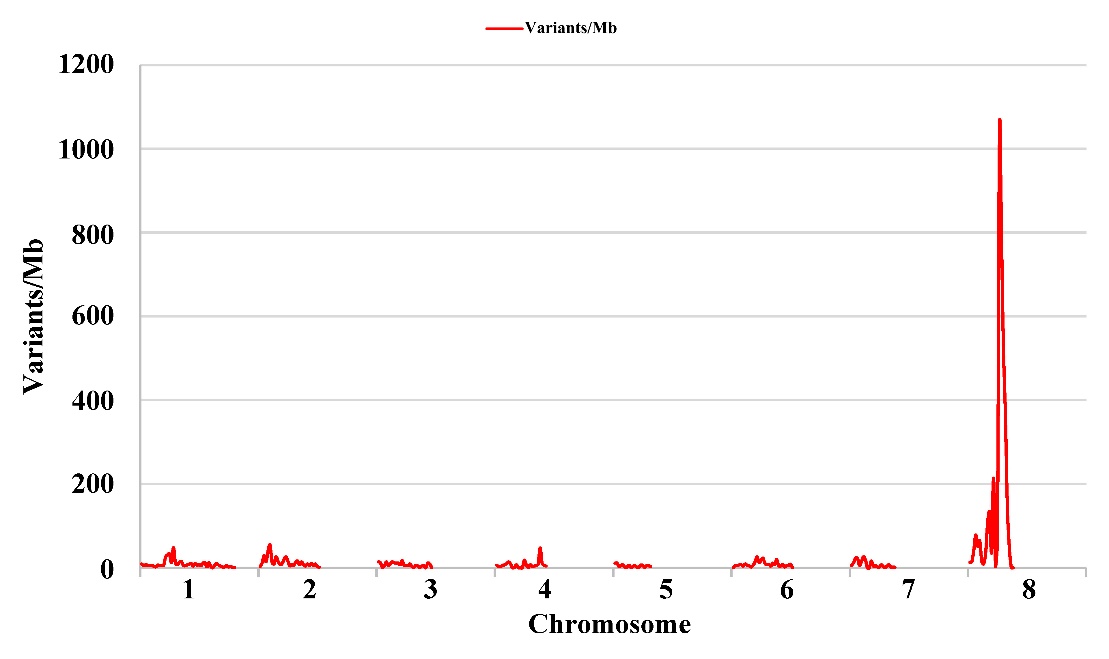


Figure S7 Distribution of density of variations associated with flower type across the eight peach chromosomes.


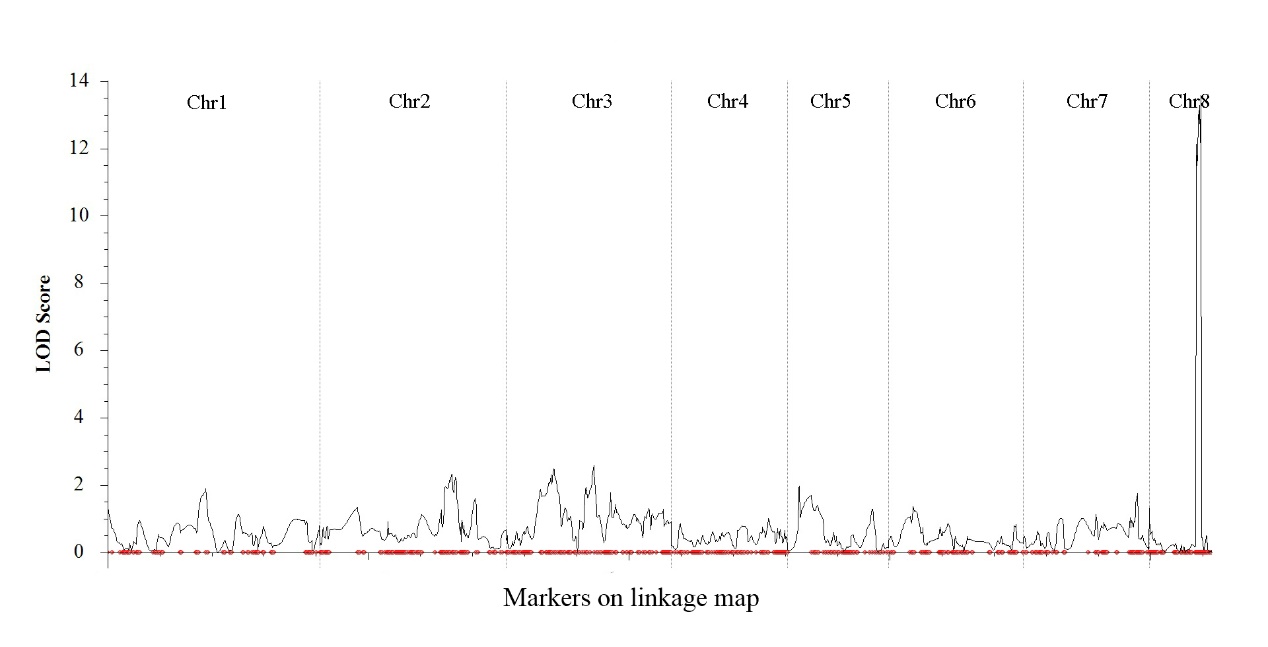


Figure S8 MTL of flower type on the genetic linkage map.


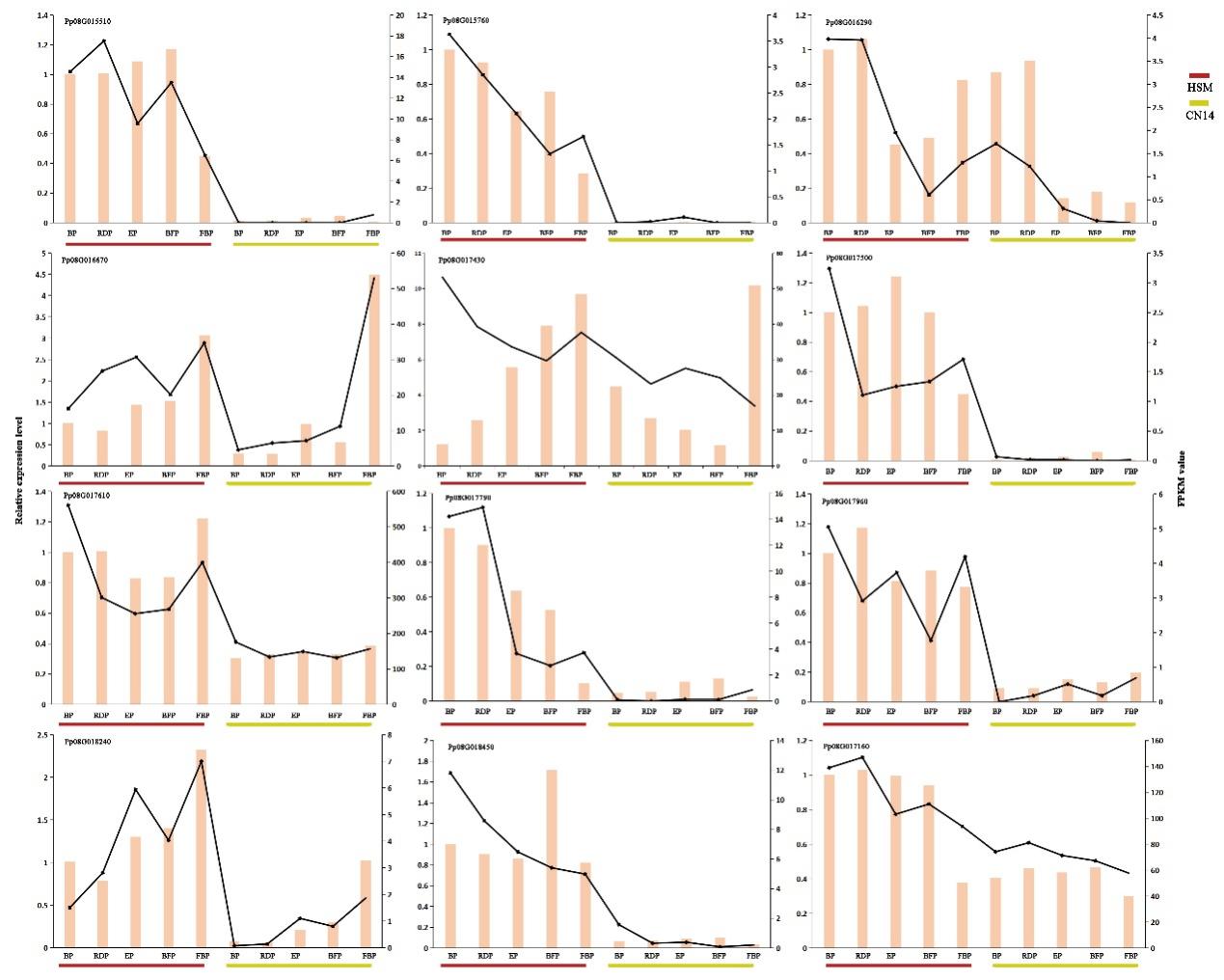


Figure S9 The relative gene expression of 15 DEGs in *SH* locus was analyzed by qRT-PCR in HSM and CN14 at five stages. The left y-axis indicates the relative gene expression; the right y-axis indicates the FPKM value.


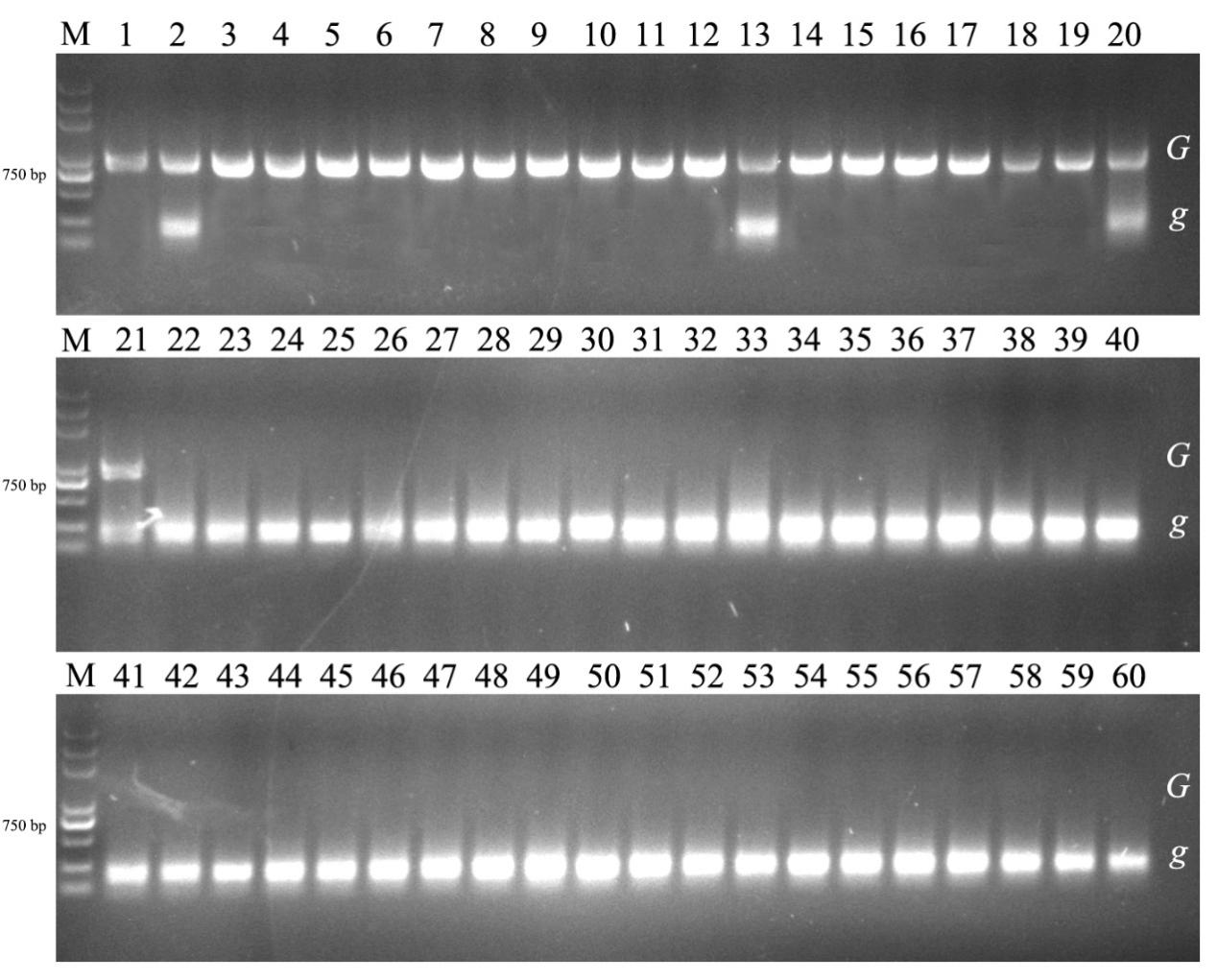


Figure S10 Identification of 6015-bp insertion of *PpMYB25* in 60 peach accessions. 1-21 showing hairiness. 22-60 showing hairless. The cultivars were indicated with numbers 1-60 as listed in Table S11. M indicated DNA Markers.


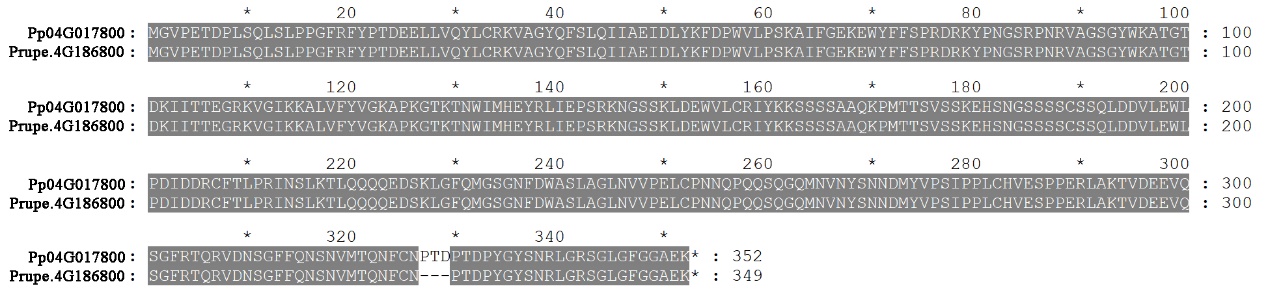


Figure S11 Sequence alignment of NAC6 in CN14 and Lovell genome. Pp04G017800 indicates a three amino acid insertion in CN14, which is an early ripening cultivar.


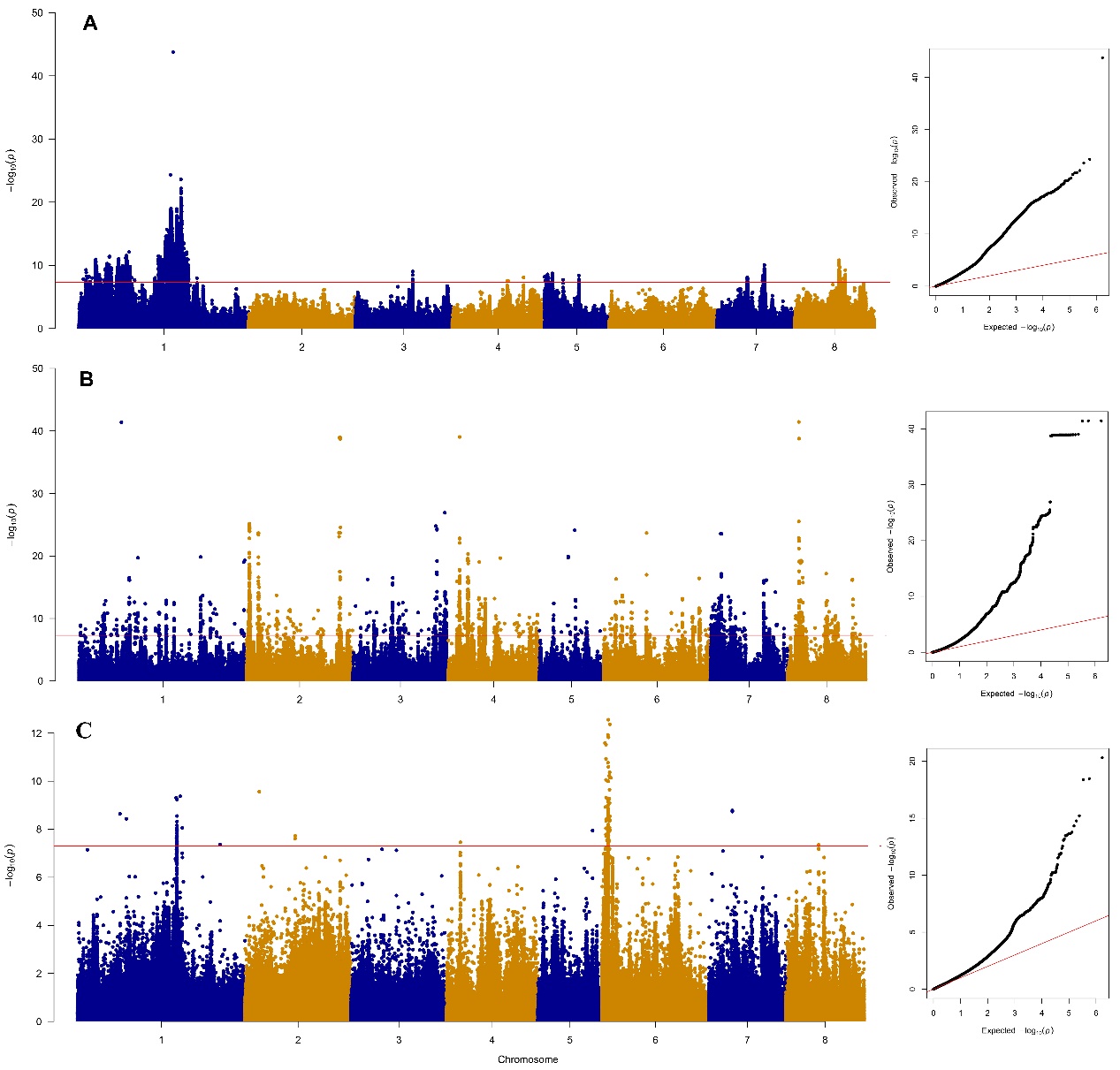


Figure S12 Identification of candidate genes for flesh color (while/ yellow), kernel taste and pollen fertility. A, A candidate locus for flesh color was located at Chr 1. B, Candidate loci for kernel taste were located at Chr 2 and Chr 8.C, A candidate locus for pollen fertility was located at Chr 6.


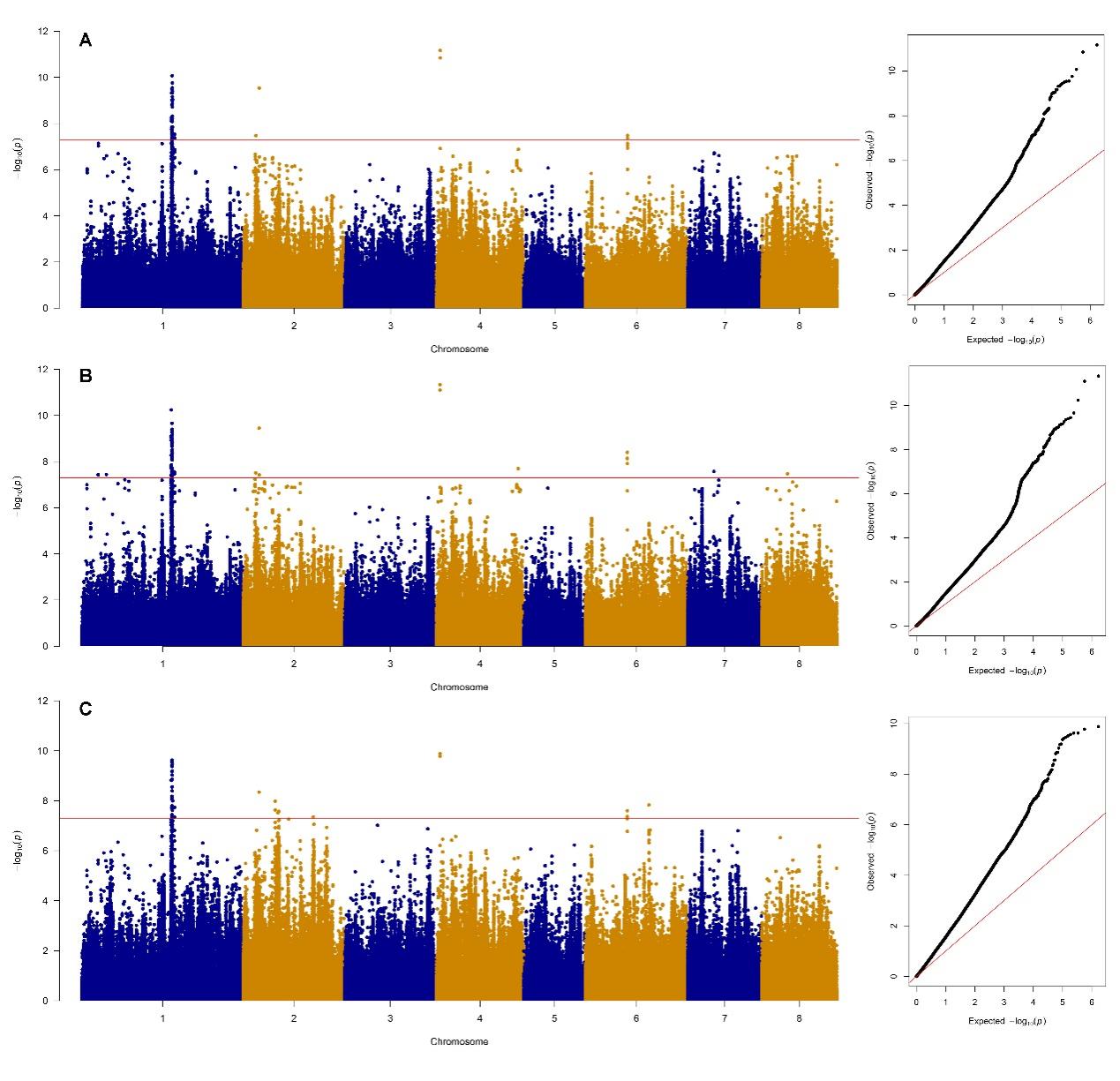


Figure S13 The same locus was associated with flower bloom date (A), full bloom date (B) and bloom ending date (C).


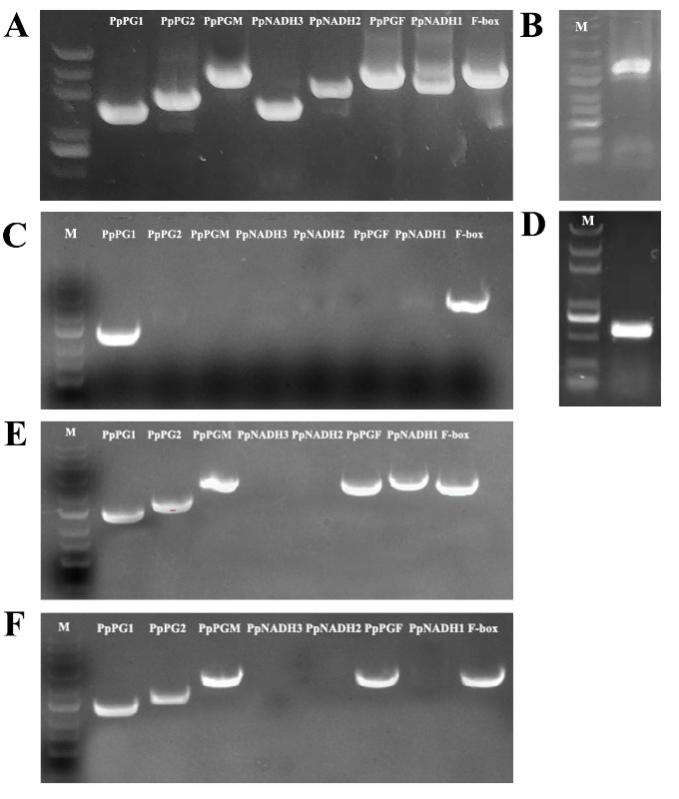


Figure S14 Agarose gel electrophoresis of the PCR products in *F-M* locus. A, PCR products of the full-length genomic DNA of eight genes in HSM. B, 12.8 Kb deletion identification using P38 primer in Everts. C, PCR products of the full-length genomic DNA of eight genes in CN14. D, 70.5 Kb deletion identification using P39 primer in CN14. E PCR products of the full-length genomic DNA of eight genes in Jinfeng. F, PCR products of the full-length genomic DNA of eight genes in Xiantao.
